# Expression of Fascin and SALL4 in odontogenic cysts and tumors: an immunohistochemical appraisal.

**DOI:** 10.12688/f1000research.126091.1

**Published:** 2022-12-23

**Authors:** Spoorti Kulkarni, Harishanker Alampally, Vasudev Guddattu, Gabriel Rodrigues, Sunitha Carnelio

**Affiliations:** 1 Oral Pathology and Microbiology, Manipal College of Dental Sciences, Manipal Academy of Higher Education (MAHE), Manipal, Karnataka, 576104, India; 2 Department of Data Science,, Prasanna School of Public Health, Manipal Academy of Higher Education, Manipal, Karnataka, 576104, India; 3 Department of General Surgery, Kasturba Medical College, Manipal Academy of Higher Education, Manipal, Karnataka, 576104, India

**Keywords:** Fascin, SALL4, ameloblastoma, immunohistochemistry, cyst, odontogenic tumor

## Abstract

**Background:** Various stemness markers (SOX2, OCT4, and NANOG) have been studied in odontogenic cysts and tumors. However, studies on SALL4 having similar properties of stemness has not been documented. Additionally, insight into fascin as a migratory molecule is less explored. In this study, the expression of SALL4 and fascin were evaluated in ameloblastoma, adenomatoid odontogenic tumor (AOT), odontogenic keratocyst (OKC), dentigerous cyst (DC), radicular cyst (RC), and calcifying odontogenic cyst (COC).

**Methods:** Semi-quantitative analysis of fascin and SALL4 immuno-positive cells was done in a total of 40 cases of ameloblastoma (11 plexiform, 12 follicular, 12 unicystic, and 5 desmoplastic) variants, 6 cases of AOT, 15 each of OKC, DC, RC and 5 of COC. Chi-square test was applied to evaluate the association between SALL4 and fascin expression in odontogenic cysts and tumors.

**Results:** Fascin immunopositivity was observed in peripheral ameloblast-like cells, and weak or absent in stellate reticulum-like cells. A moderate to weak immune-reactivity to SALL4 was observed in the cytoplasm of ameloblastoma, epithelial cells of dentigerous and radicular cysts, having a marked inflammatory infiltrate, which is an interesting observation. COC and AOT had negative to weak expressions. No recurrence has been reported.

**Conclusions:** Expression of fascin in ameloblastomas elucidate their role in motility and localized invasion. Its expression in less aggressive lesions like DC, COC, AOT will incite to explore the other functional properties of fascin. SALL4 expression in the cytoplasm of odontogenic cysts and tumors may represent inactive or mutant forms which requires further validation.

## Introduction

Odontogenic cysts and tumors are said to originate from odontogenic apparatus or oral epithelium. Ameloblastoma, the most common odontogenic tumor is known for its local but aggressive biological behaviour. ^1^ The 2017 World Health Organisation (WHO) classification on ameloblastomas have reclassified them into ameloblastoma, unicystic and extraosseous/peripheral types. ^2^ Adenomatoid odontogenic tumors (AOT) are benign with less recurrence. ^2^^,^^3^ Among the odontogenic cysts, keratocysts arising from the dental lamina are said to have aggressive behavior, appearing as multilocular radiolucencies with cortical thinning, tooth displacement and root resorption radiographically and a postoperative recurrence rate ranging from 2.5 to 62%. ^4^^,^^5^

Research to identify new markers to determine the biological behavior of odontogenic cysts and tumors is ongoing. Literature review reveals many preliminary observations with no concrete evidence of a single marker being specific to these tumors and hence there is a need to determine new markers. ^6^ In this study, we have employed two markers: fascin and SALL4. Fascin, a 55-kDa is a cytoskeleton binding proteins that bundle actin filaments, assists the cell in forming stress fibres (or ruffled borders or micro spikes) and assists cell motility and migration hence it fascin can be used for predicting the aggressive clinical course of a tumor. ^7^^–^^10^ Usually, in normal adult epithelial cells its fascin expression is low or absent. ^11^ The gene encoding fascin-1 in humans is located on chromosome 7q22. ^12^ SALL4 is a stem cell marker and a master zinc-finger transcriptional factor, as well as being a member of the spalt-like (SALL) gene family. SALL4 is mapped to chromosome 20q13.2 and plays its part in maintaining pluripotency and self-renewal of embryonic and hematopoietic stem cells by interacting with other molecules such as OCT4, SOX2 and NANOG. ^13^^–^^15^ SALL4 incorporated along with OCT4, SOX2 and KLF4 (OSK) helps in forming stable induction of pluripotent cells (iPS) cells with a higher efficiency. ^16^ Several studies noted the aberrant SALL4 expression in different types of malignant neoplasms and various autosomal dominant diseases such as Okihiro/Duane-radial ray syndrome, acro-renal-ocular syndrome, Instituto Venezolano de Investigaciones Cientificas syndrome (IVIC) and are suspected to cause thalidomide embryopathy. ^17^^–^^20^ Literature review has no reports of combination of fascin and SALL4 in odontogenic cysts and tumors to address their biological behavior and thus taking into consideration the local aggressive behavior of ameloblastoma, we hypothesise that fascin might contribute for the local migratory behavior of these odontogenic cells and SALL4 may exhibit the stemness on odontogenic cells. Thus, the aim of the present study was to evaluate the expression of fascin and SALL4 in histopathological variants of ameloblastoma, AOT and various odontogenic cysts.

## Methods

### Ethical considerations

This study was approved (IEC approval number 360/2019, 14-05-2019; IEC 156/2014, 12-03-2014) by the Institutional Ethical Committee, Kasturba Medical College, Manipal Academy of Higher Education, Manipal, India. Participant consent was waived by the committee.

### Patients and tissue samples

Formalin fixed paraffin embedded tissue (FFPE) was used for the study. Materials included a total of 40 cases of ameloblastoma (11 plexiform, 12 follicular, 12 unicystic, 5 desmoplastic) variants, 6 cases of AOT, 15 each of OKC, DC, RC and 5 of COC. Before the start of the study, statistician was consulted and based on the literature review, availability of the material in the archives of the department and the availability of the budget the sample was decided.

Samples were retrieved from the Department of Oral and Maxillofacial Pathology, Manipal College of Dental Sciences, Manipal, India after the approval from the Institutional Ethical Committee. This study used previously diagnosed samples of odontogenic cysts and tumors that were granted to us after obtaining written approval from the Head of the Department to use for this study. The samples were stored in the biobank of our department.

The diagnosis of the above said odontogenic cysts and tumors were done based on clinical and histological features (using H&E staining) according to WHO guidelines. ^2^ Sample selection was done based on inclusion and exclusion criteria: only histopathologically diagnosed cases of odontogenic cysts and tumors from 2012-2017 were considered, all the samples taken for the current study were prior to the patient receiving any treatment, cases with recurrence were excluded.

### Immunohistochemistry (IHC)

**Figure 1.**
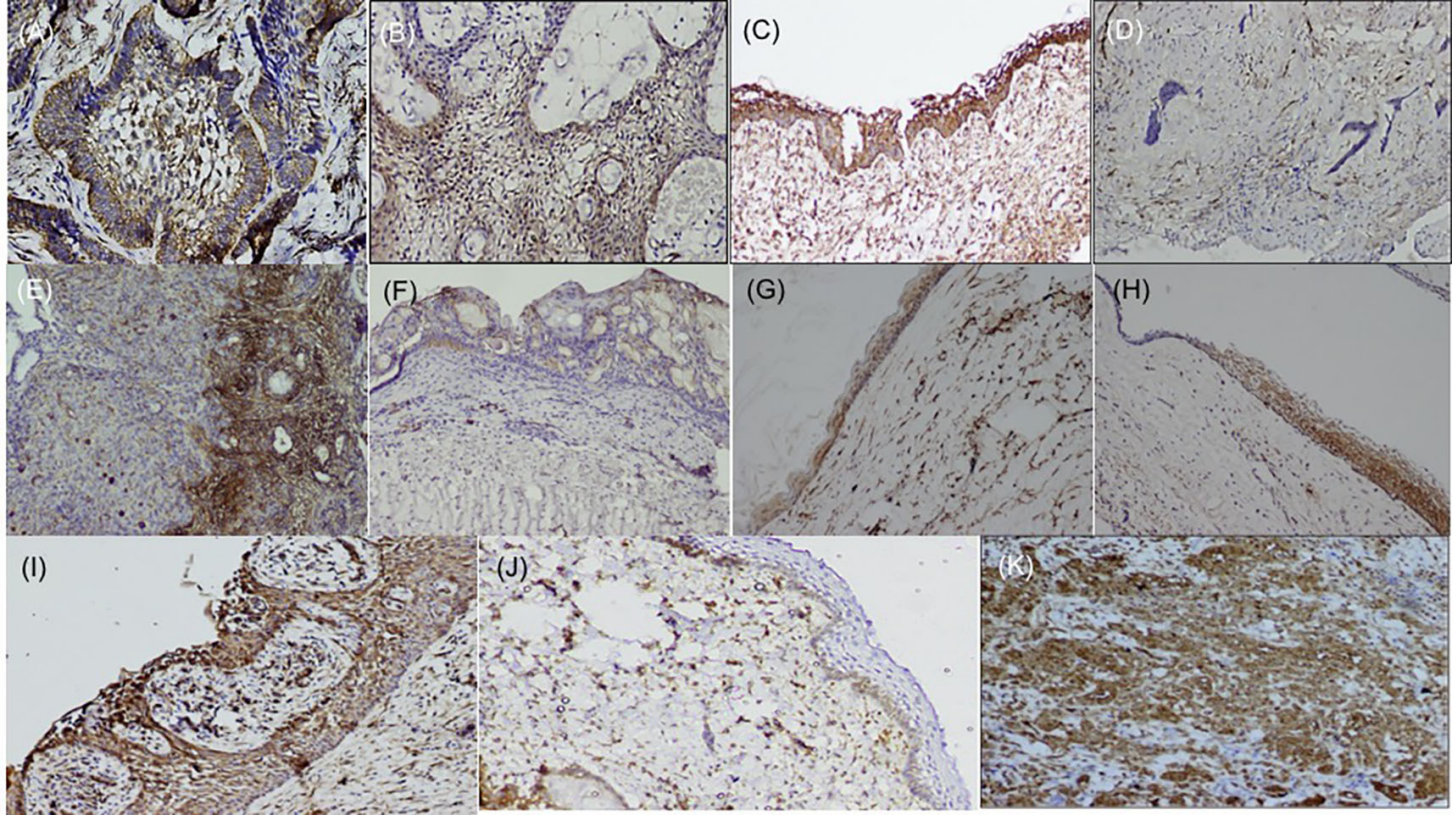
Expression of fascin in odontogenic tumors & cysts. Histopathological variants of ameloblastoma: (A) Follicular (IHC, 10×), (B) Plexiform (IHC, 10×), (C) Unicystic (IHC, 10×), (D) Desmoplastic (IHC, 4×), (E) Focal immune-positivity for fascin in AOT (IHC, 10×), (F) COC (IHC, 10×), (G) OKC (IHC, 4×), (H) Dentigerous cyst (IHC, 10×), (I) Radicular cyst, (IHC, 10×), (J) Immuno-positivity for fascin in basal cells of the oral epithelium (IHC, 4×), (K) Oral squamous cell carcinoma used as positive control stained with fascin (IHC, 10×). IHC-Immunohistochemistry. The software used record images is Olympus-DP2BSW (ver 2.1).

**Figure 2.**
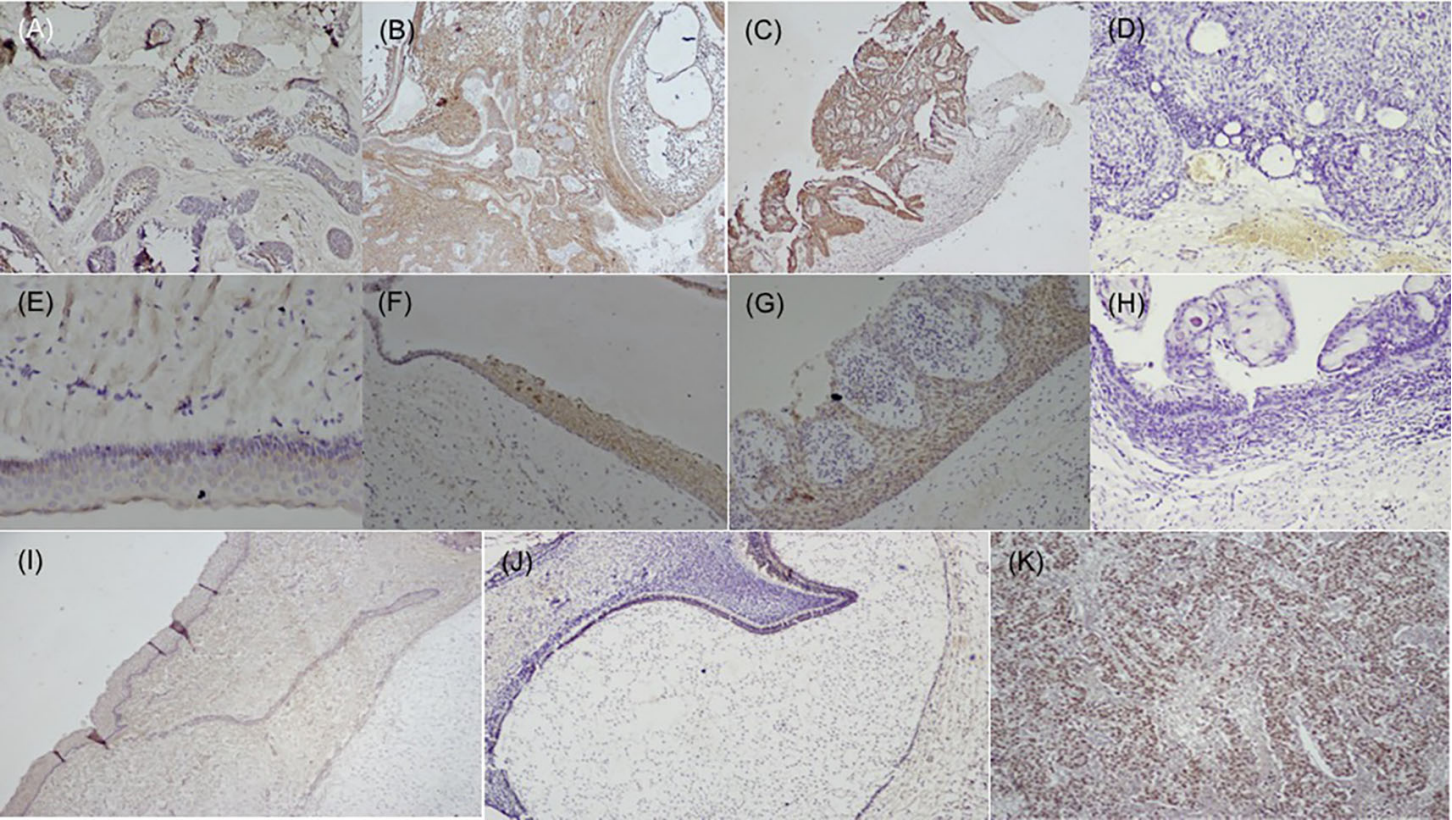
Expression of SALL4 in odontogenic cysts & tumors. Variants of ameloblastoma (A) Follicular (IHC, 10×), (B) Combination of follicular & plexiform (IHC,10×), (C) Unicystic (IHC, 4×), D) Immuno-negative in AOT (IHC, 10×), (E) OKC (IHC, 20×), (F) Dentigerous cyst (IHC, 10×), (G) Radicular cyst (IHC, 20×), (H) Immuno-negative COC (IHC, 10×), (I) Epithelial cells & ectomesenchyme surrounding the bud stage (IHC, 10×), (J) Bell stage: Focal positive to SALL4 in inner enamel epithelium (IEE) and sporadic expression in dental papilla (DP) (IHC, 10×), (K) Strong expression of SALL4 in dysgerminoma (positive control 20×). IHC-Immunohistochemistry.

Immunohistochemical staining of the tissue sections from each of the cases selected was done using the streptavidin-biotin method. In brief, 4 m sections were mounted on 3-aminopropyltriethoxysilane (APES) coated slides (Novolink Polymer Detection System, Novocastra). Sections were then deparaffinized in xylene, which is done in three grades for 10 minutes each as per standard immunohistochemical protocol. The document of the protocol has been uploaded in the repository (Open Science Framework protocol.io) ^26^ and hydrated in different grades of alcohol (ranging from absolute alcohol (10 minutes), 95 % alcohol (10 minutes), 70% (10 minutes), 50% (10 minutes) each). ^26^ Slides were then incubated with primary antibodies (rabbit monoclonal IgG for SALL4 and mouse monoclonal IgG1 for fascin) ^26^ against fascin (clone SC-21743, Santa Cruz Biotechnology USA, Inc) diluted 1:200, SALL4 (clone EP-299, PathnSitu, Livermore, USA) at a dilution of 1:100 for one hour. The sections were subsequently washed in tris-buffered saline and incubated with secondary biotinylated antibody and streptavidin-biotin peroxidase complex (Novolink Polymer Detection System, Novocastra) for 30 minutes each. Diaminobenzidine (DAB) was used as the chromogen and the sections were counterstained with Mayer’s hematoxylin. Buccal mucosa tissue was used as positive control and endothelial cells were internal controls for fascin antibody ( Figure 1), while dysgerminoma was taken as a positive control, bud and bell stage of tooth development were also included for the expression of SALL4 ( Figure 2). The primary antibody was omitted during IHC staining for the negative control.

### Immunostaining evaluation

Presence of brown color at the end of staining was considered as positive reactivity. The slides were evaluated with a light microscope (Olympus BX41) attached with Olympus DP20 microscope camera (Olympus Singapore Pvt Ltd, Singapore) at 20× & 40× magnification. The distribution of antibodies were assessed in the cytoplasm and cell membrane of ameloblastic lining of the lesions for fascin while SALL4 staining was evaluated in nuclear and cytoplasmic areas. In each case, three fields were randomly selected and evaluated.

### Staining interpretation

A semi-quantitative method was used to score the fascin and SALL4 expression in the epithelial odontogenic cells.

Based on intensity: (a) of the immunostaining in the epithelial odontogenic cells (0-1 = absent/weak, 2 = moderate, 3 = strong).

Degree of staining: (b) the percentage of positive odontogenic cells (1 ≤ 25% positive cells, 2 = 25-50% positive, 3 = 51-75% positive and 4 ≥ 75% positive cells).

Total staining: The final immunostaining score was determined by the sum of (a) + (b). Final scores ranged from 0 to 7 (0 = absent, 1-4 = weak and 5-7 = strong).

### Statistical analysis

The data obtained was statistically analyzed with the statistical software program SPSS (version 17.0). The statistical significance of fascin and SALL4 in histopathological types of ameloblastoma was analysed using the chi-square test. *P* values less than 0.05 were considered to indicate statistical significance.

## Results

Immunohistochemically stained sections of various odontogenic cysts and tumor tissue were evaluated for expression of SALL4 in the cytoplasm as well as nuclei of peripheral ameloblastic cells and stellate reticulum like cells while expression of fascin was observed in the cell membrane, between cell boundaries and cytoplasm of peripheral ameloblastic cells, stellate reticulum like cells and stromal cells of 40 cases of ameloblastoma variants from the year 2012 to 2017. The expression of fascin and SALL4 varied from case to case as well as in the same tissue section. Most of the variants of ameloblastoma were strongly positive for fascin but cases of desmoplastic ameloblastoma (5/5) were negative for fascin ( Table 1A, Figure 1D). To eliminate bias, two observers independently evaluated the expression of these, selecting the most representative site separately under a light microscope at 200× and 400× magnification. The SALL4 positivity was heterogeneous with varied intensity and staining pattern with absence of expression in few follicles ( Figure 2) but in most of the follicular, plexiform and unicystic ameloblastoma, the immunopositivity was observed diffuse in the cytoplasm and less localised to the nucleus ( Figure 2A– C) but the stromal cells were devoid of its expression except in the endothelial cells. Fascin expression was found to be weak or absent in stellate reticulum like cells ( Figure 1). In cases of unicystic ameloblastoma, positivity for fascin was observed in the basal as well as in the upper layers ( Figure 1C). However intra-group comparison did not show any significant difference. The final scoring was obtained after combining the intensity and percentage scoring and was found to be strong in most of the cases ( Table 1A).

**Table 1A.** SALL4 and fascin in odontogenic tumors.

		UA		PA		FA		DA		AOT		Total	X ^2^	P value	Significance
Distribution of odontogenic tumors	Females	6		4		4		3		2		19	1.67	0.79	NS
	Males	6		7		8		2		4		27			
Sores of staining intensity		S	F	S	F	S	F	S	F	S	F	SAL	31.5	<0.001	S
	1 (Weak)	0	0	0	0	0	0	5	5	4	5				
	2 (Moderate)	4	2	5	2	5	2	0	0	2	1	FSN			
	3 (Strong)	8	10	6	9	7	10	0	0	0	0				
Scores of stained cell count	1 (0-25%)	0	1	0	0	0	1	5	5	4	2	SAL			
	2 (26-50%)	2	0	0	0	0	1	0	0	2	4				
	3 (51-75%)	8	2	3	2	3	4	0	0	0	0				
	4 (76-100%)	2	9	8	9	9	6	0	0	0	0	FSN			
IRS	Absent/Weak (0-4)	2	1	0	0	0	1	5	5	6	6				
	Strong (5-7)	10	11	11	11	12	11	0	0	0	0				

UA: Unicystic Ameloblastoma, PA: Plexiform Ameloblastoma, FA: Follicular Ameloblastoma, DA: Desmoplastic Ameloblastoma, AOT: Adenomatoid Odontogenic Tumor, SAL: SALL4, FSN: Fascin, S: Significant, NS: Non significant, IRS: Immunoreactive score.

AOT was immune-positive to fascin in few areas (< 25%) with mild to moderate intensity ( Figure 1E), while immune-negative for SALL4 expression ( Figure 2D). Fascin expression in odontogenic cysts (OKC, RC, DC) ( Figure 1G– I) was strongly positive with greater than 75% cells, while intensity ranged from moderate to strong along the cystic lining. COC revealed immune-positivity ranging from 25-50% ( Figure 1F). SALL4 expression in odontogenic cysts was strongly positive with greater than 75% cells stained, while intensity ranged from mild to moderate with a diffuse cytoplasmic staining, at places nuclear staining was evident ( Table 1B), Figure 2E– G). COC was immune-negative ( Figure 2H).

**Table 1B.** SALL4 and fascin in odontogenic cyst.

		COC		OKC		DC		RC		Total	X ^2^	P value	Significance
Distribution of odontogenic cyst	Females	2		3		3		5		13	1.49	0.68	NS
	Males	3		12		12		10		37			
Sores of staining intensity		S	F	S	F	S	F	S	F	SAL	8.01	0.091	NS
	1 (Weak)	5	3	5	0	6	0	0	0				
	2 (Moderate)	0	2	9	4	9	0	11	4	FSN			
	3 (Strong)	0	0	1	11	0	15	4	11				
Scores of stained cell count	1 (0-25%)	4	3	0	0	1	0	0	0	SAL			
	2 (26-50%)	1	1	3	2	2	0	0	0				
	3 (51-75%)	0	1	7	1	2	4	4	2				
	4 (76-100%)	0	0	5	12	10	11	11	13	FSN			
IRS	Absent/Weak (0-4)	5	5	7	0	3	0	0	0				
	Strong (5-7)	0	0	8	15	12	15	15	15				

COC: Calcifying Odontogenic Cyst, OKC: Odontogenic Keratocyst DC: Dentigerous cyst, RC: Radicular cyst, SAL: SALL4, FSN: Fascin, S: Significant, IRS: Immunoreactive score.

## Discussion

Researchers have worked on the molecular mechanism to understand the nature of local invasion of ameloblastomas into the surrounding tissues which include molecules degrading the extracellular matrix, those involved in bone remodelling, molecules associated with angiogenesis and molecules related to proliferation. Though the results are partially promising, the exact molecular mechanism of invasion in ameloblastomas is not completely understood. ^6^ Cell motility is essential for tumor invasion and subsequent dissemination or metastases. This increase in motility occurs via the modulation of actin filaments to form finger-like plasma membrane protrusions termed invadopodia. Numerous actin-binding proteins, including fascin, regulate such dynamic rearrangement of the actin cytoskeleton. Fascin, being one of the actin cross-linking proteins, localizes to filopodia at the leading edge of migratory cells by organising f-actin into well-ordered, tightly packed parallel bundles observed *in vitro* studies. ^21^

Fascin overexpression is observed in various precancerous lesions and oral squamous cell carcinoma (OSCC). ^9^^,^^12^ In our study, we observed that a majority of our cases were strongly positive for fascin in the various subtypes of ameloblastoma. Various *in vitro* and *in vivo* studies have observed that fascin has a functional role in cell invasion and motility. ^13^ This could account to the local aggressiveness of ameloblastoma clinically. Few of the ameloblastic follicles did not exhibit fascin, we speculate this could be attributed to loss of antigen during processing or reduced motility in these cells.

In various histopathological grades of ameloblastoma, SALL4 was expressed in the majority of cases. Studies have documented transcription activity of SALL4, which could be reflected by its positivity in the nucleus. ^15^^,^^22^ We observed that the odontogenic epithelial cells were positive for SALL4 in the cytoplasm, stained diffusely, which we speculate could be in an inactive/dormant or mutant form which requires further investigation. Majority of OKC were devoid of SALL4 except in the basal cells. Radicular and dentigerous cysts, having marked infiltration of inflammatory cells had strong immune-positivity for SALL4 in the cytoplasm, an interesting finding of this study. Hence the role of cytokines in stimulating SALL4 needs to be ruled out. Odontogenic tumors, AOT and developmental odontogenic cysts, COC (simple type) were negative for SALL4. Studies have shown that OKCs expressed higher amount of PCNA and Ki-67 when compared to other jaw cysts, indicating its inherently increased proliferative potential of OKC. ^23^ This speculates that various other molecular pathways could play an important role in the disease process. Further studies are required to explore this possibility, since this is a preliminary study.

Normal connective tissue cells such as fibroblasts, vascular endothelial cells, neural and glial cells, brain and splenic tissue expressed fascin, which relates to its function, required to maintain normal homeostasis. ^9^ In embryogenesis, various migratory cells express fascin, except in terminally differentiated squamous cells where its expression is low or absent. ^9^^,^^24^ Our previous study on tooth buds showed fascin expression in various stages of tooth development was site and time specific, thus confirming its role in cell remodulation. ^3^ SALL4 expression was not detected in tooth bud stage, however focal positivity was observed in the cytoplasm of the epithelial cells of bell stage, this could attribute to the cells to undergo more differentiated state of the cells. ^25^ The papillary cells in various stages of tooth germ were positive ( Figure 2J) and this could relate to stemness due to the pluripotency nature of dental papilla. Further studies are required to understand the crosstalk with other stem cell markers in maintaining the stemness or pluripotency state of the cells.

In conclusion, the findings of the present study on the expression of fascin elucidate their role in motility and localized invasion or in maintaining the cellular homeostasis, while the expression of SALL4 remains elusive.

## Data availability

Open Science Framework: Expression of fascin and SALL4 in odontogenic cysts and tumors: an immunohistochemical appraisal. https://doi.org/10.17605/OSF.IO/9ZFRS. ^26^

Data are available under the terms of the Creative Commons Zero “No rights reserved” data waiver (CC0 1.0 Public domain dedication).

## References

[1] Effiom OA Ogundana OM Akinshipo AO : Ameloblastoma: current etiopathological concepts and management. *Oral Dis.* 2018;24:307–316. 10.1111/odi.12646 28142213

[2] Reichart P Sciubba JJ Philipsen HP : Splitters or lumpers: The 2017 WHO Classification of Head and Neck Tumors. *J. Am. Dent. Assoc.* 2018;149:567–571. 10.1016/j.adaj.2018.03.029 29754695

[3] Alampally H Chandrashekar C Rodrigues G : Fascin in tooth germs: an immunohistochemical analysis. *J. Histotechnol.* 2018;41:24–28. 10.1080/01478885.2017.1404286

[4] Rajendra Santosh AB : Odontogenic cysts. *Dent. Clin. N. Am.* 2020;64:105–119. 10.1016/j.cden.2019.08.002 31735221

[5] Bilodeau EA Collins BM : Odontogenic cysts and neoplasms. *Surg. Pathol. Clin.* 2017;10:177–222. 10.1016/j.path.2016.10.006 28153133

[6] Júnior JF França GM de Silva Barros CC da : Biomarkers involved in the proliferation of the odontogenic keratocyst, glandular odontogenic cyst and botryoid odontogenic cyst. *Oral Maxillofac. Surg.* 2022 Jan 21; 10.1007/s10006-021-01026-x 35059898

[7] Lamb MC Tootle TL : Fascin in cell migration: more than an actin bundling protein. *Biology (Basel).* 2020;9:403. 33212856 10.3390/biology9110403 PMC7698196

[8] Yamashiro S : Functions of fascin in dendritic cells. *Crit. Rev. Immunol.* 2012;32:11–22. 10.1615/CritRevImmunol.v32.i1.20 22428853

[9] Lamptey J Czika A Aremu JO : The role of fascin in carcinogenesis and embryo implantation. *Exp. Cell Res.* 2021;409:112885. 10.1016/j.yexcr.2021.112885 34662557

[10] Adams JC : Fascin protrusions in cell interactions. *Trends Cardiovasc. Med.* 2004;14:221–226. 10.1016/j.tcm.2004.06.002 15451513

[11] Zhang X Cho IH Park JH : Fascin is involved in cancer cell invasion and is regulated by stromal factors. *Oncol. Rep.* 2019;41:465–474. 10.3892/or.2018.6847 30542700

[12] Liu H Zhang Y Li L : Fascin actin-bundling protein 1 in human cancer: promising biomarker or therapeutic target? *Mol. Ther. Oncolytics.* 2021;20:240–264. 10.1016/j.omto.2020.12.014 33614909 PMC7873579

[13] Xiong J : SALL4: engine of cell stemness. *Curr. Gene Ther.* 2014;14:400–411. 10.2174/1566523214666140825125138 25174577 PMC13202290

[14] Zhang X Yuan X Zhu W : SALL4: an emerging cancer biomarker and target. *Cancer Lett.* 2015;357:55–62. 10.1016/j.canlet.2014.11.037 25444934

[15] Tatetsu H Kong NR Chong G : SALL4, the missing link between stem cells, development and cancer. *Gene.* 2016;584:111–119. 10.1016/j.gene.2016.02.019 26892498 PMC4823161

[16] Parchem RJ Ye J Judson RL : Two miRNA clusters reveal alternative paths in late-stage reprogramming. *Cell Stem Cell.* 2014;14:617–631. 10.1016/j.stem.2014.01.021 24630794 PMC4305531

[17] Kohlhase J Chitayat D Kotzot D : SALL4 mutations in Okihiro syndrome (Duane-radial ray syndrome), acro-renal-ocular syndrome, and related disorders. *Hum. Mutat.* 2005;26:176–183. 10.1002/humu.20215 16086360

[18] Borozdin W Boehm D Leipoldt M : SALL4 deletions are a common cause of Okihiro and acro-renal-ocular syndromes and confirm haploinsufficiency as the pathogenic mechanism. *J. Med. Genet.* 2004;41:e113. 10.1136/jmg.2004.019901 15342710 PMC1735888

[19] Paradisi I Arias S : IVIC syndrome is caused by a c.2607delA mutation in the SALL4 locus. *Am. J. Med. Genet. A.* 2007;143:326–332. 17256792 10.1002/ajmg.a.31603

[20] Gao S Wang S Fan R : Recent advances in the molecular mechanism of thalidomide teratogenicity. *Biomed. Pharmacother.* 2020;127:110114. 10.1016/j.biopha.2020.110114 32304852

[21] Pfisterer K Levitt J Lawson CD : FMNL2 regulates dynamics of fascin in filopodia. *J. Cell Biol.* 2020;219:e201906111. 10.1083/jcb.201906111 32294157 PMC7199847

[22] Yang J Corsello TR Ma Y : Stem cell gene SALL4 suppresses transcription through recruitment of DNA methyltransferases. *J. Biol. Chem.* 2012;287:1996–2005. 10.1074/jbc.M111.308734 22128185 PMC3265879

[23] Kaplan I Hirshberg A : The correlation between epithelial cell proliferation and inflammation in odontogenic keratocyst. *Oral Oncol.* 2004;40:985–991. 10.1016/j.oraloncology.2004.04.017 15509489

[24] De Arcangelis A Georges-Labouesse E Adams JC : Expression of fascin-1, the gene encoding the actin-bundling protein fascin-1, during mouse embryogenesis. *Gene Expr. Patterns.* 2004;4:637–643. 10.1016/j.modgep.2004.04.012 15465486

[25] Rodas-Junco BA Villicaña C : Dental pulp stem cells: current advances in isolation, expansion and preservation. *Tissue Eng. Regen. Med.* 2017;14:333–347. 10.1007/s13770-017-0036-3 30603490 PMC6171610

[26] Kulkarni S Alampally H Guddattu V : Expression of fascin and SALL4 in odontogenic cysts and tumors: An immunohistochemical appraisal. *OSF.* 12 Sept. 2022. 10.17605/OSF.IO/9ZFRS https://osf.io/9zfrs/ PMC11184278 38895097

