# Expression of Fascin and SALL4 in odontogenic cysts and tumors: an immunohistochemical appraisal.

**DOI:** 10.12688/f1000research.126091.3

**Published:** 2023-12-14

**Authors:** Spoorti Kulkarni, Harishanker Alampally, Vasudev Guddattu, Gabriel Rodrigues, Sunitha Carnelio

**Affiliations:** 1 Oral Pathology and Microbiology, Manipal College of Dental Sciences, Manipal Academy of Higher Education (MAHE), Manipal, Karnataka, 576104, India; 2 Department of Data Science, Prasanna School of Public Health, Manipal Academy of Higher Education, Manipal, Karnataka, 576104, India; 3 Department of General Surgery, Kasturba Medical College, Manipal Academy of Higher Education, Manipal, Karnataka, 576104, India

**Keywords:** Fascin, SALL4, Ameloblastoma, Immunohistochemistry, Odontogenic cysts, Odontogenic tumors

## Abstract

**Background:**

Various stemness markers (SOX2, OCT4, and NANOG) have been studied in odontogenic cysts and tumors. However, studies on SALL4 having similar properties of stemness has not been documented. Additionally, insight into fascin as a migratory molecule is less explored. In this study, the expression of SALL4 and fascin were evaluated in ameloblastoma, adenomatoid odontogenic tumor (AOT), odontogenic keratocyst (OKC), dentigerous cyst (DC), radicular cyst (RC), and calcifying odontogenic cyst (COC).

**Methods:**

Semi-quantitative analysis of fascin and SALL4 immuno-positive cells was done in a total of 40 cases of ameloblastoma (11 plexiform, 12 follicular, 12 unicystic, and 5 desmoplastic) variants, 6 cases of AOT, 15 each of OKC, DC, RC and 5 of COC. Chi-square test was applied to evaluate the association between SALL4 and fascin expression in odontogenic cysts and tumors.

**Results:**

Fascin immunopositivity was observed in peripheral ameloblast-like cells, and weak or absent in stellate reticulum-like cells. A moderate to weak immune-reactivity to SALL4 was observed in the cytoplasm of ameloblastoma, epithelial cells of dentigerous and radicular cysts, having a marked inflammatory infiltrate, which is an interesting observation. COC and AOT had negative to weak expressions. No recurrence has been reported.

**Conclusions:**

Expression of fascin in ameloblastomas elucidate their role in motility and localized invasion. Its expression in less aggressive lesions like DC, COC, AOT will incite to explore the other functional properties of fascin. SALL4 expression in the cytoplasm of odontogenic cysts and tumors may represent inactive or mutant forms which requires further validation.

## Introduction

Odontogenic cysts and tumors are said to originate from odontogenic apparatus or oral epithelium. Ameloblastoma, the most common odontogenic tumor is known for its local but aggressive biological behaviour. ^1^ The 2017 World Health Organisation (WHO) classification on ameloblastomas have reclassified them into Conventional, Unicyctic and Peripheral ^2^ Literature review states among the odontogenic lesions, Ameloblastoma and Odontogenic keratocyst are locally aggressive and recurrent lesions, also the commonest and prevalent odontogenic tumor in Indian population is ameloblastoma which ranges from 14.02% to 71.4% when compared to other odontogenic tumors. ^3^ ^,^ ^4^ The globally, pooled estimate of the incidence rate of ameloblastoma is 0.92 per million population per year. ^5^ The recurrence varies among various populations, 9.8% according to a Chinese study, ^6^ while in European multicenter study it is reported to be 19.3%, ^7^also tumors larger than 6 cm and involving the soft tissues or adjacent anatomical structures are associated with early recurrence irrespective of method of surgery. Also conservatively (marsupialization, enucleation, curettage) treated cases have a high recurrence rate compared to radical treatment. ^6^ However there is no concrete data pertaining recurrence on AOT, they are benign with rare recurrence. The other odontogenic cysts included were developmental viz DC and COC, wherein DC is associated with an impacted tooth while COC is associated with calcifications and ghost epithelial cells. RC an inflammatory odontogenic cyst is commonly associated with carious or non-vital tooth. ^2^

Research to identify new markers to determine the biological behavior of odontogenic cysts and tumors is ongoing. Literature review reveals many preliminary observations with no concrete evidence of a single marker being specific to these tumors and hence there is a need to determine new markers. ^6^ In this study, we have employed two markers: fascin and SALL4. Fascin, a 55-kDa is a cytoskeleton binding protein that bundle actin filaments, assists the cell in forming stress fibres (or ruffled borders or micro spikes) and assists cell motility and migration hence fascin can be used for predicting the aggressive clinical course of a tumor. ^7^ ^–^ ^10^ Usually, in normal adult epithelial cells fascin expression is low or absent. ^11^ The gene encoding fascin-1 in humans is located on chromosome 7. SALL4 is a stem cell marker and a master zinc-finger transcriptional factor, and a member of the spalt-like (SALL) gene family. ^12^ SALL4 is mapped to chromosome 20q13.2 and plays its part in maintaining pluripotency and self-renewal of embryonic and hematopoietic stem cells by interacting with other molecules such as OCT4, SOX2 and NANOG. ^13^ ^–^ ^15^ SALL4 incorporated along with OCT4, SOX2 and KLF4 (OSK) helps in forming stable induction of pluripotent cells (iPS) cells with a higher efficiency. ^16^ Several studies noted the aberrant SALL4 expression in different types of malignant neoplasms and various autosomal dominant diseases such as Okihiro/Duane-radial ray syndrome, acro-renal-ocular syndrome, Instituto Venezolano de Investigaciones Cientificas syndrome (IVIC) and are suspected to cause thalidomide embryopathy. ^17^ ^–^ ^20^ Literature review confirms fascin contributes for cell motility and migration in many studies (Pubmed 376 articles), SALL4 contributes to stemness along with other stem cell markers (SOX2, OCT4, and NANOG). ^8^ ^–^ ^13^ Studies have shown the expression of these stem cell markers in Ameloblastoma & OKC except SALL4. ^14^ Most of the studies in SALL4 are related to malignant soft tissue tumors, ^15^ ^,^ ^16^ no reports are available of SALL4 expression in odontogenic lesions. SALL4 is activated by various pathways such as Wnt/β-catenin, ^15^ PI3K/AKT, signalling pathway through targeting PTEN ^16^ or Notch signalling pathway ^17^ thus facilitating migration, invasion and proliferation, while Fascin is activated via PI3K/ Akt pathway _._ ^18^Also literature reports cross talk between Wnt/β-catenin and PI3K/Akt pathways or simultaneous activation of these pathways contributing for proliferation and cell migration. ^19^ ^–^ ^21^ Hence the present study was done to evaluate and compare the expression of these two biomarkers in various odontogenic tumors (Histopathological variants of Ameloblastoma, AOT) and odontogenic cysts (OKC,DC,RC,COC).

## Methods

### Patients and tissue samples

Formalin fixed paraffin embedded tissue (FFPE) sections were retrieved from the Department of Oral and Maxillofacial Pathology, Manipal College of Dental Sciences, Manipal, India after obtaining approval from Institutional Ethical Committee, (IEC approval number 360/2019, IEC 156/2014). The samples taken up for the study were from year 2012-2017 which included 40 cases of ameloblastoma with histopathological variants viz plexiform (no:11), follicular (no:12), Unicystic (no:12), desmoplastic (no.5),6 cases of AOT, 15 cases each of OKC, DC, RC and 5 cases of COC. The cases selected did comply to inclusion and exclusion criteria. All the samples taken for the current study were prior to the patient receiving any treatment, cases with recurrence were excluded. The diagnosis of the above said odontogenic cysts and tumors were based on clinical and histological features (using H&E staining) according to WHO guidelines. ^2^

### Immunohistochemistry (IHC)

**Figure 1.**
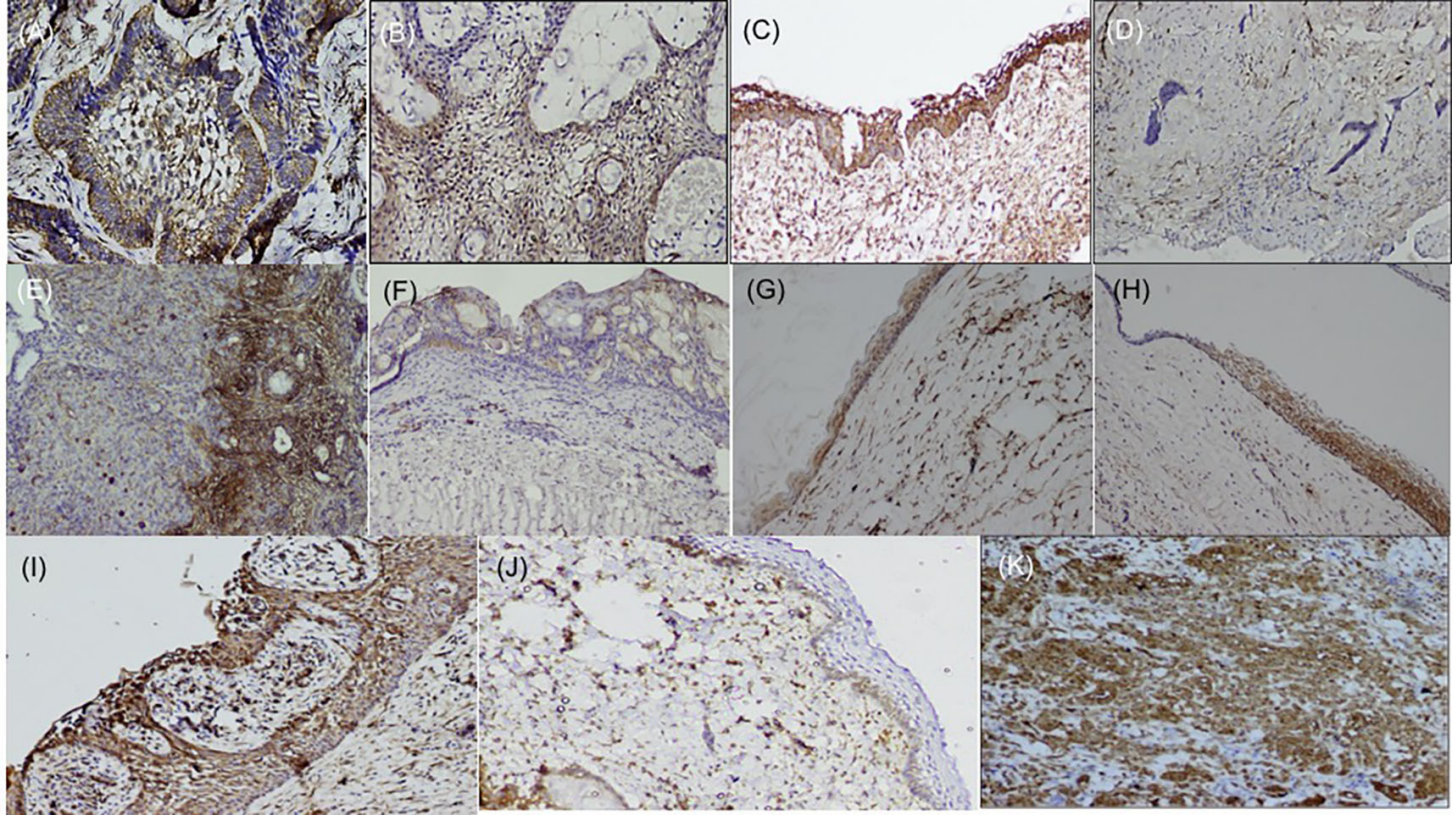
Expression of fascin in odontogenic tumors & cysts. Histopathological variants of ameloblastoma: (A) Follicular (IHC, 10×), (B) Plexiform (IHC, 10×), (C) Unicystic (IHC, 10×), (D) Desmoplastic (IHC, 4×), (E) Focal immune-positivity for fascin in AOT (IHC, 10×), (F) COC (IHC, 10×), (G) OKC (IHC, 4×), (H) Dentigerous cyst (IHC, 10×), (I) Radicular cyst, (IHC, 10×), (J) Immuno-positivity for fascin in basal cells of the oral epithelium (IHC, 4×), (K) Oral squamous cell carcinoma used as positive control stained with fascin (IHC, 10×). IHC-Immunohistochemistry. The software used record images is Olympus-DP2BSW (ver 2.1).

**Figure 2.**
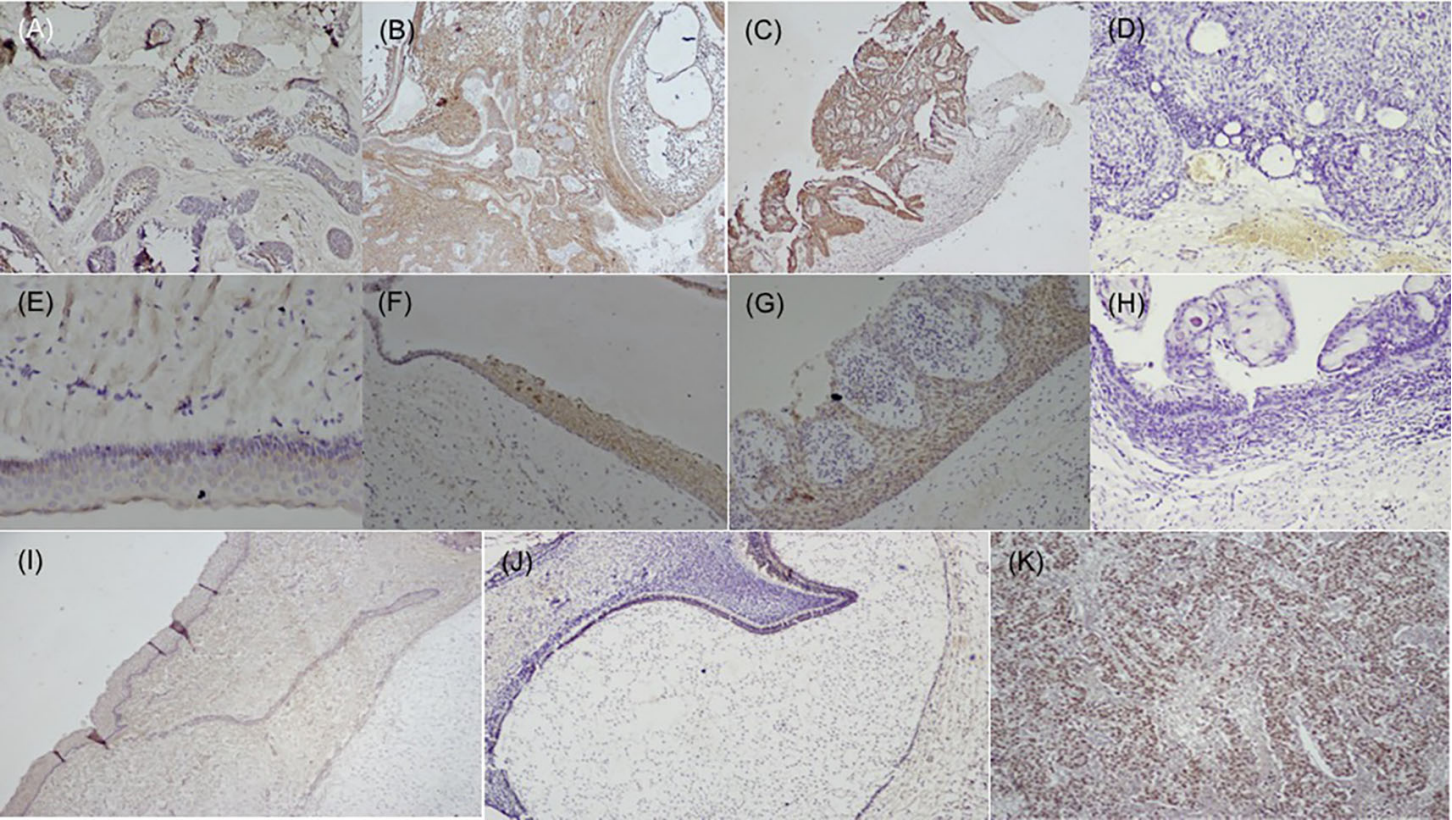
Expression of SALL4 in odontogenic cysts & tumors. Variants of ameloblastoma (A) Follicular (IHC, 10×), (B) Combination of follicular & plexiform (IHC,10×), (C) Unicystic (IHC, 4×), D) Immuno-negative in AOT (IHC, 10×), (E) OKC (IHC, 20×), (F) Dentigerous cyst (IHC, 10×), (G) Radicular cyst (IHC, 20×), (H) Immuno-negative COC (IHC, 10×), (I) Epithelial cells & ectomesenchyme surrounding the bud stage (IHC, 10×), (J) Bell stage: Focal positive to SALL4 in inner enamel epithelium (IEE) and sporadic expression in dental papilla (DP) (IHC, 10×), (K) Strong expression of SALL4 in dysgerminoma (positive control 20×). IHC-Immunohistochemistry.

Immunohistochemical staining of the tissue sections from each of the cases selected was done using the streptavidin-biotin method. In brief, 4 *μ*m sections were mounted on 3-aminopropyltriethoxysilane (APES) coated slides (Novolink Polymer Detection System, Novocastra). Sections were then deparaffinized in xylene, which was done in three grades for 10 minutes and hydrated in different grades of alcohol ranging from absolute alcohol (10 minutes), 95 % alcohol (10 minutes), 70% (10 minutes), 50% (10 minutes) each. Sections were then incubated with primary antibodies, rabbit antihuman SALL4 monoclonal antibody at a dilution of 1:100(IgG, clone EP-299, PathnSitu, Livermore, USA), mouse antihuman fascin monoclonal antibody (IgG1,clone 55K-2, SC-21743, Santa Cruz Biotechnology USA, Inc) diluted at 1:200. The sections were subsequently washed in tris-buffered saline and incubated with secondary biotinylated antibody and streptavidin-biotin peroxidase complex (Novolink Polymer Detection System, Novocastra) for 30 minutes each. Diaminobenzidine (DAB) was used as the chromogen and the sections were counterstained with Mayer’s hematoxylin. Buccal mucosa tissue was used as positive control, ^22^ the basal cells of the epithelium were stained positive and endothelial cells within the lesional tissue were internal controls for fascin antibody ( Figure 1), while dysgerminoma was taken as a positive control, for SALL4, for positive nuclear expression ( Figure 2). Bud and bell stage of tooth development were also included for the study. The primary antibody was replaced during IHC staining for the negative control as per standard immunohistochemical protocol. The document of the protocol has been uploaded in the repository (Open Science Framework protocol.io). ^48^

### Immunostaining evaluation

Presence of brown color at the end of staining was considered as positive reactivity. The slides were evaluated with a light microscope (Olympus BX41) attached with Olympus DP20 microscope camera (Olympus Singapore Pvt Ltd, Singapore) at 20× & 40× magnification. The distribution of antibodies was assessed in the cytoplasm and cell membrane of ameloblastic lining of the lesions for fascin while SALL4 staining was evaluated in nuclear areas. In each case, three fields were randomly selected, and two observers independently evaluated the expression of these biomarkers, after selecting the most representative site separately under a light microscope at 200× and 400× magnification to eliminate the bias.

### Staining interpretation

A semi-quantitative method was used to score the fascin and SALL4 expression in the epithelial odontogenic cells.

Based on intensity: (a) of the immunostaining in the epithelial odontogenic cells (0-1 = absent/weak, 2 = moderate, 3 = strong).

Degree of staining: (b) the percentage of positive odontogenic cells (1 ≤ 25% positive cells, 2 = 25-50% positive, 3 = 51-75% positive and 4 ≥ 75% positive cells).

Total staining: The final immunostaining score was determined by the sum of (a) + (b). Final scores ranged from 0 to 7 (0 = absent, 1-4 = weak and 5-7 = strong).

### Statistical analysis

The data obtained was statistically analyzed with the statistical software program SPSS (version 17.0). The statistical significance of fascin and SALL4 in histopathological types of ameloblastoma was analysed using the chi-square test. *P* values less than 0.05 were considered to indicate statistical significance.

## Results

Immunohistochemically stained sections of various odontogenic cysts and tumors were evaluated for expression of fascin in the cell membrane, between cell boundaries and cytoplasm of peripheral ameloblastic cells, stellate reticulum like cells and stromal cells of 40 cases of ameloblastoma variants while expression of SALL4 was observed in the cytoplasm as well as nuclei of peripheral ameloblastic cells and stellate reticulum like cells. The total IRS score was the main outcome ( Table 1, Table 2). Chi-square test was used to compare the frequency of distribution of categorized total IRS score with fascin and SALL4 in various odontogenic tumors (Ameloblastoma and its histopathological variants, AOT) and odontogenic cysts (OKC, DC, COC, RC). The expression of fascin and SALL4 varied from case to case as well as in the same tissue section. Most of the variants of ameloblastoma were strongly positive for fascin but cases of desmoplastic ameloblastoma (5/5) were negative for fascin ( Figure 1D). Fascin expression was found to be weak or absent in stellate reticulum like cells ( Figure 1). In cases of unicystic ameloblastoma, positivity for fascin was observed in the basal as well as in the suprabasal layers ( Figure 1C). However intra-group comparison did not show any significant difference. AOT was immune-positive to fascin in few areas (< 25%) with mild to moderate intensity ( Figure 1E). Fascin expression in odontogenic cysts (OKC, RC, DC) ( Figure 1G–I) was strongly positive with greater than 75% cells, while intensity ranged from moderate to strong along the cystic lining. COC revealed immune positivity ranging from 25-50% ( Figure 1F). The SALL4 positivity was heterogeneous with varied intensity and staining pattern. In most of the histopathological variant of ameloblastoma, the immunopositivity observed, was diffuse in the cytoplasm and less localised to the nucleus ( Figure 2A–C). The stromal cells were devoid of its expression except in the endothelial cells. SALL4 expression in odontogenic cysts was strongly positive with greater than 75% cells exhibiting diffuse cytoplasmic staining. Nuclear staining was evident in few cells ( Figure 2E–G). COC was immune-negative ( Figure 2H).

**Table 1.** Expression of Fascin and SALL4 in odontogenic tumors.

Odontogenic tumor (n=52)	Frequency (n,%)	Total IRS score	SALL4	Fascin	X ^2^	P	S/NS
Ameloblastoma	n=46 (88.46%)						
	Subtypes						
	UA (n=12)	Absent/Weak (0-4)	2	0	0.38	1	NS
		Strong (5-7)	10	12			
	PA (n=11)	Absent/Weak (0-4)	0	0	-	1	NS
		Strong (5-7)	11	11			
	FA (n=12)	Absent/Weak (0-4)	0	1	1.04	1	NS
		Strong (5-7)	12	11			
	DA (n=5)	Absent/Weak (0-4)	5	5	-	1	NS
		Strong (5-7)	0	0			
AOT	6 (11.54%)	Absent/Weak (0-4)	6	6	-	1	NS
		Strong (5-7)	0	0			

UA, Unicystic Ameloblastoma; PA, Plexiform Ameloblastoma; FA, Follicular Ameloblastoma; DA, Desmoplastic Ameloblastoma; AOT, Adenomatoid Odontogenic Tumor; p-value, Probability value; n, frequency; IRS, Immunoreactive score; Total IRS Score=Score of staining intensity + Scores of stained cell count.

**Table 2.** Expression of Fascin and SALL4 in odontogenic cysts.

Odontogenic cyst (n=50)	Frequency (n,%)	Total IRS score	SALL4	Fascin	X ^2^	P	S/NS
COC	5(10%)	Absent/Weak (0-4)	5	5	-	1	NS
		Strong (5-7)	0	0			
OKC	15(30%)	Absent/Weak (0-4)	7	0	9.1	0.006	NS
		Strong (5-7)	8	15			
DC	15(30%)	Absent/Weak (0-4)	3	0	3.33	0.22	NS
		Strong (5-7)	12	15			
RC	15(30%)	Absent/Weak (0-4)	0	0	-	1	NS
		Strong (5-7)	15	15			

COC, Calcifying Odontogenic Cyst; OKC, Odontogenic Keratocyst; DC, Dentigerous cyst; RC, Radicular cyst; P-value, Probability value; n, frequency; IRS Score=Score of staining intensity + Scores of stained cell count.

Regarding the evaluation of the statistical significance test, in all the odontogenic tumors, the staining intensity of fascin was similar compared to SALL4. With regard to the stained cell count, higher counts were observed with fascin as compared to SALL4. In relation to odontogenic cysts, OKC and DC, the intensity of fascin was more than SALL4. Also the higher cell counts were observed in fascin as compared to SALL4 in odontogenic keratocyst. The data regarding the same has been attached as Supplementary files(S1,S2,S3,S4) and has been uploaded in the repository (open science framework).

## Discussion

Researchers have worked on the molecular mechanism to understand the nature of local invasion of ameloblastomas into the surrounding tissues which include molecules degrading the extracellular matrix, those involved in bone remodelling, molecules associated with angiogenesis and molecules related to proliferation. ^23^ Though the results are partially promising, the exact molecular mechanism of invasion in ameloblastomas is not completely understood. ^23^ ^–^ ^27^ Cell motility is essential for tumor invasion and subsequent dissemination or metastases. This increase in motility occurs via the modulation of actin filaments to form finger-like plasma membrane protrusions termed invadopodia. Numerous actin-binding proteins, including fascin, regulate such dynamic rearrangement of the actin cytoskeleton. Fascin, being one of the actin cross-linking proteins, localizes to filopodia at the leading edge of migratory cells by organising f-actin into well-ordered, tightly packed parallel bundles observed *in vitro* studies. ^28^

Fascin overexpression is observed in various precancerous lesions and oral squamous cell carcinoma (OSCC). ^29^ ^–^ ^33^ In our study, we observed that a majority of our cases were strongly positive for fascin in the various subtypes of ameloblastoma. Various *in vitro* and *in vivo* studies have observed that fascin has a functional role in cell invasion and motility. ^34^ This could account to the local aggressiveness of ameloblastoma clinically. Few of the ameloblastic follicles did not exhibit fascin, we speculate this could be attributed to loss of antigen during processing or reduced motility in these cells. Fascin expression in various cysts such as DC,OKC,RC and COC could be related to its influence in focal adhesion and cell dynamics. ^35^

In various histopathological grades of ameloblastoma, SALL4 was expressed in the majority of cases. Studies have documented transcription activity of SALL4, which could be reflected by its positivity in the nucleus. ^36^ ^–^ ^44^ We observed that the odontogenic epithelial cells were positive for SALL4 in the cytoplasm, stained diffusely, which we speculate could be in an inactive/dormant or mutant form which requires further investigation. Majority of OKC were devoid of SALL4 except in the basal cells. Radicular and dentigerous cysts, having marked infiltration of inflammatory cells had strong immune-positivity for SALL4 in the cytoplasm, an interesting finding of this study. Hence the role of cytokines in stimulating SALL4 needs to be ruled out. Odontogenic tumors, AOT and developmental odontogenic cysts, COC (simple type) were negative for SALL4. Studies have shown that OKCs expressed higher amount of PCNA and Ki-67 when compared to other jaw cysts, indicating its inherently increased proliferative potential of OKC. ^45^ This speculates that various other molecular pathways could play an important role in the disease process. Further studies are required to explore this possibility, since this is a preliminary study.

Normal connective tissue cells such as fibroblasts, vascular endothelial cells, neural and glial cells, brain and splenic tissue expressed fascin, which relates to its function, required to maintain normal homeostasis. ^30^ In embryogenesis, various migratory cells express fascin, except in terminally differentiated squamous cells where its expression is low or absent. ^30^ ^,^ ^46^ Our previous study on tooth buds showed fascin expression in various stages of tooth development was site and time specific, thus confirming its role in cell remodulation. ^3^ SALL4 expression was not detected in tooth bud stage, however focal positivity was observed in the cytoplasm of the epithelial cells of bell stage, this could attribute to the cells to undergo more differentiated state of the cells. ^47^ The papillary cells in various stages of tooth germ were positive ( Figure 2J) and this could relate to stemness due to the pluripotency nature of dental papilla. Further experimental validation to elucidate their functional significance needs is required to understand the crosstalk with other stem cell markers in maintaining the stemness or pluripotency state of the cells.

In conclusion, the findings of the present study on the expression of fascin elucidate their role in motility and localized invasion or in maintaining the cellular homeostasis, while the expression of SALL4 remains elusive.

## Data availability

Open Science Framework: Expression of fascin and SALL4 in odontogenic cysts and tumors: an immunohistochemical appraisal. https://doi.org/10.17605/OSF.IO/9ZFRS. ^48^

Data are available under the terms of the Creative Commons Zero “No rights reserved” data waiver (CC0 1.0 Public domain dedication).

## References

[1] Effiom OA Ogundana OM Akinshipo AO : Ameloblastoma: current etiopathological concepts and management. *Oral Dis.* 2018;24:307–316. 10.1111/odi.12646 28142213

[2] Reichart P Sciubba JJ Philipsen HP : Splitters or lumpers: The 2017 WHO Classification of Head and Neck Tumors. *J. Am. Dent. Assoc.* 2018;149:567–571. 10.1016/j.adaj.2018.03.029 29754695

[3] Ahire MS Tupkari JV Chettiankandy TJ : Odontogenic tumors: A 35-year retrospective study of 250 cases in an Indian (Maharashtra) teaching institute. *Indian journal of cancer.* 2018 Jul 1;55(3):265–272. 10.4103/ijc.IJC_145_18 30693892

[4] Pandiar D Shameena PM Sudha S : Odontogenic Tumors: A 13-year Retrospective Study of 395 Cases in a South Indian Teaching Institute of Kerala. *Oral & Maxillofacial Pathology Journal.* 2015 Jul 1;6(2).

[5] Hendra FN Van Cann EM Helder MN : Global incidence and profile of ameloblastoma: a systematic review and meta-analysis. *Oral Diseases.* 2020 Jan;26(1):12–21. 10.1111/odi.13031 30614154

[6] Yang R Liu Z Gokavarapu S : Recurrence and cancerization of ameloblastoma: multivariate analysis of 87 recurrent craniofacial ameloblastoma to assess risk factors associated with early recurrence and secondary ameloblastic carcinoma. *Chinese Journal of Cancer Research.* 2017 Jun;29(3):189–195. 10.21147/j.issn.1000-9604.2017.03.04 28729769 PMC5497205

[7] Boffano P Cavarra F Tricarico G : The epidemiology and management of ameloblastomas: A European multicenter study. *Journal of Cranio-Maxillofacial Surgery.* 2021 Dec 1;49(12):1107–1112. 10.1016/j.jcms.2021.09.007 34583885

[8] Matoba R Niwa H Masui S : Dissecting Oct3/4-regulated gene networks in embryonic stem cells by expression profiling. *PLoS One.* 2006 Dec 20;1(1): e26. 10.1371/journal.pone.0000026 17183653 PMC1762406

[9] Nishimoto M Fukushima A Okuda A : The gene for the embryonic stem cell coactivator UTF1 carries a regulatory element which selectively interacts with a complex composed of Oct-3/4 and Sox-2. *Molecular and cellular biology.* 1999 Aug 1;19(8):5453–5465. 10.1128/MCB.19.8.5453 10409735 PMC84387

[10] Wang J Rao S Chu J : A protein interaction network for pluripotency of embryonic stem cells. *Nature.* 2006 Nov 16;444(7117):364–368. 10.1038/nature05284 17093407

[11] Wu Q Chen X Zhang J : Sall4 interacts with Nanog and co-occupies Nanog genomic sites in embryonic stem cells. *Journal of Biological Chemistry.* 2006 Aug 25;281(34):24090–24094. 10.1074/jbc.C600122200 16840789

[12] Zhang J Tam WL Tong GQ : Sall4 modulates embryonic stem cell pluripotency and early embryonic development by the transcriptional regulation of Pou5f1. *Nature cell biology.* 2006 Oct 1;8(10):1114–1123. 10.1038/ncb1481 16980957

[13] Zhou Q Chipperfield H Melton DA : A gene regulatory network in mouse embryonic stem cells. *Proceedings of the National Academy of Sciences.* 2007 Oct 16;104(42):16438–16443. 10.1073/pnas.0701014104 17940043 PMC2034259

[14] Phattarataratip E Panitkul T Khodkaew W : Expression of SOX2 and OCT4 in odontogenic cysts and tumors. *Head & Face Medicine.* 2021 Dec;17:1–7. 10.1186/s13005-021-00283-1 34261507 PMC8278639

[15] Zhang X Zhong N Li X : TRIB3 promotes lung cancer progression by activating β-catenin signaling. *European Journal of Pharmacology.* 2019 Nov 15;863: 172697. 10.1016/j.ejphar.2019.172697 31562867

[16] Jiang G Liu CT : Knockdown of SALL4 overcomes cisplatin-resistance through AKT/mTOR signaling in lung cancer cells. *International Journal of Clinical and Experimental Pathology.* 2018;11(2):634–641. 31938149 PMC6958024

[17] Park JT Chen X Tropè CG : Notch3 overexpression is related to the recurrence of ovarian cancer and confers resistance to carboplatin. *The American journal of pathology.* 2010 Sep 1;177(3):1087–1094. 10.2353/ajpath.2010.100316 20671266 PMC2928943

[18] Fleming-de-Moraes CD Rocha MR Tessmann JW : Crosstalk between PI3K/Akt and Wnt/β-catenin pathways promote colorectal cancer progression regardless of mutational status. *Cancer biology & therapy.* 2022 Dec 31;23(1):1–3. 10.1080/15384047.2022.2108690 35944058 PMC9367664

[19] Laguë MN Paquet M Fan HY : Synergistic effects of Pten loss and WNT/CTNNB1 signaling pathway activation in ovarian granulosa cell tumor development and progression. *Carcinogenesis.* 2008 Nov 1;29(11):2062–2072. 10.1093/carcin/bgn186 18687666 PMC2577137

[20] Deming DA Leystra AA Nettekoven L : PIK3CA and APC mutations are synergistic in the development of intestinal cancers. *Oncogene.* 2014 Apr;33(17):2245–2254. 10.1038/onc.2013.167 23708654 PMC3883937

[21] Pearson HB Phesse TJ Clarke AR : K-ras and Wnt signaling synergize to accelerate prostate tumorigenesis in the mouse. *Cancer research.* 2009 Jan 1;69(1):94–101. 10.1158/0008-5472.CAN-08-2895 19117991

[22] Rodrigues PC Sawazaki-Calone I Ervolino de Oliveira C : Fascin promotes migration and invasion and is a prognostic marker for oral squamous cell carcinoma. *Oncotarget.* 2017;8(43):74736–74754. 10.18632/oncotarget.20360 29088820 PMC5650375

[23] Fuchigami T Ono Y Kishida S : Molecular biological findings of ameloblastoma. *Jpn Dent Sci Rev.* 2021 Nov;57:27–32. 10.1016/j.jdsr.2020.12.003 33737992 PMC7946346

[24] Alampally H Chandrashekar C Rodrigues G : Fascin in tooth germs: an immunohistochemical analysis. *Journal of Histotechnology.* 2018;41:24–28. 10.1080/01478885.2017.1404286

[25] Rajendra Santosh AB : Odontogenic cysts. *Dent Clin North Am.* 2020;64:105–119. 10.1016/j.cden.2019.08.002 31735221

[26] Bilodeau EA Collins BM : Odontogenic cysts and neoplasms. *Surg Pathol Clin.* 2017;10:177–222. 10.1016/j.path.2016.10.006 28153133

[27] Júnior JF França GM de Silva Barros CC da : Biomarkers involved in the proliferation of the odontogenic keratocyst, glandular odontogenic cyst and botryoid odontogenic cyst. *Oral Maxillofac Surg.* 2022 Jan 21;26:655–662. 10.1007/s10006-021-01026-x 35059898

[28] Lamb MC Tootle TL : Fascin in cell migration: more than an actin bundling protein. *Biology (Basel).* 2020;9:403. 33212856 10.3390/biology9110403 PMC7698196

[29] Yamashiro S : Functions of fascin in dendritic cells. *Crit Rev Immunol.* 2012;32:11–22. 10.1615/CritRevImmunol.v32.i1.20 22428853

[30] Lamptey J Czika A Aremu JO : The role of fascin in carcinogenesis and embryo implantation. *Exp Cell Res.* 2021;409: 112885. 10.1016/j.yexcr.2021.112885 34662557

[31] Adams JC : Fascin protrusions in cell interactions. *Trends Cardiovasc Med.* 2004;14:221–226. 10.1016/j.tcm.2004.06.002 15451513

[32] Zhang X Cho IH Park JH : Fascin is involved in cancer cell invasion and is regulated by stromal factors. *Oncol Rep.* 2019;41:465–474. 10.3892/or.2018.6847 30542700

[33] Liu H Zhang Y Li L : Fascin actin-bundling protein 1 in human cancer: promising biomarker or therapeutic target? *Mol Ther Oncolytics.* 2021;20:240–264. 10.1016/j.omto.2020.12.014 33614909 PMC7873579

[34] Xiong J : SALL4: engine of cell stemness. *Curr Gene Ther.* 2014;14:400–411. 10.2174/1566523214666140825125138 25174577 PMC13202290

[35] Villari G Jayo A Zanet J : A direct interaction between fascin and microtubules contributes to adhesion dynamics and cell migration. *J Cell Sci.* 2015;128(24):4601–4614. 10.1242/jcs.175760 26542021 PMC4696496

[36] Zhang X Yuan X Zhu W : SALL4: an emerging cancer biomarker and target. *Cancer Lett.* 2015;357:55–62. 10.1016/j.canlet.2014.11.037 25444934

[37] Tatetsu H Kong NR Chong G : SALL4, the missing link between stem cells, development and cancer. *Gene.* 2016;584:111–119. 10.1016/j.gene.2016.02.019 26892498 PMC4823161

[38] Parchem RJ Ye J Judson RL : Two miRNA clusters reveal alternative paths in late-stage reprogramming. *Cell Stem Cell.* 2014;14:617–631. 10.1016/j.stem.2014.01.021 24630794 PMC4305531

[39] Kohlhase J Chitayat D Kotzot D : SALL4 mutations in Okihiro syndrome (Duane-radial ray syndrome), acro-renal-ocular syndrome, and related disorders. *Hum Mutat.* 2005;26:176–183. 10.1002/humu.20215 16086360

[40] Borozdin W Boehm D Leipoldt M : SALL4 deletions are a common cause of Okihiro and acro-renal-ocular syndromes and confirm haploinsufficiency as the pathogenic mechanism. *J. Med. Genet.* 2004;41: e113. 10.1136/jmg.2004.019901 15342710 PMC1735888

[41] Paradisi I Arias S : IVIC syndrome is caused by a c.2607delA mutation in the SALL4 locus. *Am J Med Genet A.* 2007;143:326–332. 10.1002/ajmg.a.31603 17256792

[42] Gao S Wang S Fan R : Recent advances in the molecular mechanism of thalidomide teratogenicity. *Biomed Pharmacother.* 2020;127: 110114. 10.1016/j.biopha.2020.110114 32304852

[43] Pfisterer K Levitt J Lawson CD : FMNL2 regulates dynamics of fascin in filopodia. *J Cell Biol.* 2020;219: e201906111. 10.1083/jcb.201906111 32294157 PMC7199847

[44] Yang J Corsello TR Ma Y : Stem cell gene SALL4 suppresses transcription through recruitment of DNA methyltransferases. *J. Biol. Chem.* 2012;287:1996–2005. 10.1074/jbc.M111.308734 22128185 PMC3265879

[45] Kaplan I Hirshberg A : The correlation between epithelial cell proliferation and inflammation in odontogenic keratocyst. *Oral Oncol.* 2004;40:985–991. 10.1016/j.oraloncology.2004.04.017 15509489

[46] De Arcangelis A Georges-Labouesse E Adams JC : Expression of fascin-1, the gene encoding the actin-bundling protein fascin-1, during mouse embryogenesis. *Gene Expr. Patterns.* 2004;4:637–643. 10.1016/j.modgep.2004.04.012 15465486

[47] Rodas-Junco BA Villicaña C : Dental pulp stem cells: current advances in isolation, expansion and preservation. *Tissue Eng. Regen. Med.* 2017;14:333–347. 10.1007/s13770-017-0036-3 30603490 PMC6171610

[48] Kulkarni S Alampally H Guddattu V : Expression of fascin and SALL4 in odontogenic cysts and tumors: An immunohistochemical appraisal. *OSF.* 12 Sept. 2022. 10.17605/OSF.IO/9ZFRS PMC11184278 38895097

