# Supplementary material for: Expression of Fascin and SALL4 in odontogenic cysts and tumors: an immunohistochemical appraisal
Source: F1000Res. 2024 Jun 27;11:1578. Originally published 2022 Dec 23. [Version 5] doi: 10.12688/f1000research.126091.5 (PMC11184278; doi:10.12688/f1000research.126091.5)
Supplement: Peer review history

# Reviewer report: Reviewer response for version 5

**DOI:** 10.5256/f1000research.168274.r296320

**Published:** 2024-07-03

**Authors:** Pentti Nieminen

**Affiliations:** 1 Medical Informatics and Statistics Research Group, University of Oulu, Oulu, Finland

Authors have responded to my requests. I have no further comment to make.

Is the work clearly and accurately presented and does it cite the current literature?

No

If applicable, is the statistical analysis and its interpretation appropriate?

No

Are all the source data underlying the results available to ensure full reproducibility?

Partly

Is the study design appropriate and is the work technically sound?

No

Are the conclusions drawn adequately supported by the results?

Partly

Are sufficient details of methods and analysis provided to allow replication by others?

No

Reviewer Expertise:

Statistical reporting and data presentation

I confirm that I have read this submission and believe that I have an appropriate level of expertise to confirm that it is of an acceptable scientific standard.

# Reviewer report: Reviewer response for version 4

**DOI:** 10.5256/f1000research.167388.r281189

**Published:** 2024-06-18

**Authors:** Hope M Amm

**Affiliations:** 1 The University of Alabama at Birmingham, Birmingham, Alabama, USA

The manuscript “Expression of Fascin and SALL4 in odontogenic cysts and tumors: an immunohistochemical appraisal” examines the expression of these markers in odontogenic tumors. However, there are some details lacking and the data could be presented more clearly.

1. The authors spend a portion of the introduction telling what pathways regulate fascin and SALL4. While fascin and SALL4 have not been previously reported in odontogenic tumors, some of these pathways have. This relationship should be included in the discussion.
2. Please include how long tissues were incubated with the primary antibody as it has relevance to repeatability.
3. The authors state “Buccal mucosa tissue was used as a positive control…” Would not this mean these markers are poor biomarkers for odontogenic tumors if they are generally expressed in the oral cavity.
4. Figures 1 and 2 should be more standardized in appearance and presentation. Some images are darker than others making it difficult to see what is positive. The samples should be presented in the same order and same magnification in each figure for ease of comparison and clarity (i.e., A-D. 4 forms of ameloblastoma, E. AOT, F. COC, G. OKC, H. DC, I. RC, J-K. Positive controls.
5. Desmoplatic ameloblastoma should be included in Figure 2.
6. Unicystic ameloblastoma is better represented by 4x (Figure 2) than 10x (Figure 1).
7. Figure 2E does not appear positive as stated and is not clear.
8. The expression of fascin looks quite strong in Figure 1E.  Were multiple images used in the pathologic evaluation?
9. Literature has not shown any clinical significance to histologic variants. A comparison of unicystic ameloblastoma to conventional ameloblastoma would be more relevant.
10. In the results there is mention of SALL4 nuclear staining. The magnifications shown are too low to see this in the figures presented. Perhaps include an offset at a higher magnification where you wish to demonstrate nuclear staining.
11. For fascin to be acting as described in the discussion it would need to be staining more organized (with the actin cytoskeleton) rather than diffusely. Please relate your discussion items to your data more clearly.

Is the work clearly and accurately presented and does it cite the current literature?

Yes

If applicable, is the statistical analysis and its interpretation appropriate?

Yes

Are all the source data underlying the results available to ensure full reproducibility?

Yes

Is the study design appropriate and is the work technically sound?

Partly

Are the conclusions drawn adequately supported by the results?

Partly

Are sufficient details of methods and analysis provided to allow replication by others?

Yes

Reviewer Expertise:

Odontogenic tumors

I confirm that I have read this submission and believe that I have an appropriate level of expertise to state that I do not consider it to be of an acceptable scientific standard, for reasons outlined above.

# Response

**Published:** 2024-06-22

**Authors:** Dr. Spoorti Kulkarni

**Affiliations:** Manipal College of Dental Sciences, Manipal Academy of Higher Education, Manipal, India

We thank Dr. Hope M Amm for reviewing our article and providing valuable feedback. We have carefully considered and addressed the comments.

**Reviewer Q1:** The authors spend a portion of the introduction telling what pathways regulate fascin and SALL4. While fascin and SALL4 have not been previously reported in odontogenic tumors, some of these pathways have. This relationship should be included in the discussion.

**Author Response:** The present study evaluated the immuno-expression of two markers Fascin and SALL4.  A detailed findings of the current study were discussed. We did emphasize in the discussion the pathways to be explored concerning these markers.

 “In the current study, one of the limitations was the small sample size. Studies with a larger sample size are needed to understand the molecular ( fascin and SALL4)  dynamics, functional behavior which is required for further validation and unravel the interaction of the pathways that activate these molecules. The interactivity of these molecules with other stem cell molecules in maintaining the stemness or pluripotency state of the cells needs to be explored.”

**Reviewer Q2:** Please include how long tissues were incubated with the primary antibody as it has relevance to repeatability.

 Response: The incubation time for SALL4 with the primary antibody was for 60 minutes at room temperature and the incubation time for fascin with primary antibody was 30 minutes at room temperature, as per the manufacturer's protocol. The same has been incorporated in the methodology.

**Reviewer Q3:** The authors state “Buccal mucosa tissue was used as a positive control…” Would not this mean these markers are poor biomarkers for odontogenic tumors if they are generally expressed in the oral cavity.

**Author Response:** Tooth buds and buccal mucosa were taken as controls also the markers (SALL4& Fascin) employed for the current study are not specific for odontogenic tumors. Moreover, the basal cell of the surface epithelium is one of the proposed pathogenesis contributing to ameloblastomas( Reference: Sivapathasundharam B. Shafer's Textbook of Oral Pathology. 9th ed. St. Louis: Elsevier; 2020.

**Reviewer Q4:** Figures 1 and 2 should be more standardized in appearance and presentation. Some images are darker than others making it difficult to see what is positive. The samples should be presented in the same order and same magnification in each figure for ease of comparison and clarity (i.e., A-D. 4 forms of ameloblastoma, E. AOT, F. COC, G. OKC, H. DC, I. RC, J-K. Positive controls.

**Author Response: **We have tried to capture the most representative field with suitable magnification for this study.

**Reviewer Q5:** Desmoplastic ameloblastoma should be included in Figure 2.

 Response: Figure 2 illustrates the expression of the marker SALL4 in various odontogenic cysts and tumors.

**Reviewer Q6: **Unicystic ameloblastoma is better represented by 4x (Figure 2) than 10x (Figure 1).

**Author Response:** Thank you, we appreciate your suggestions.

**Reviewer Q7:** Figure 2E does not appear positive as stated and is not clear.

**Author Response:** SALL4 positivity was appreciated in a few cells and mostly in the cytoplasm.

**Reviewer Q8:** The expression of fascin looks quite strong in Figure 1E.  Were multiple images used in the pathologic evaluation?

**Author Response:** Yes, multiple images were used for evaluation.

**Reviewer Q9: **Literature has not shown any clinical significance to histologic variants. A comparison of unicystic ameloblastoma to conventional ameloblastoma would be more relevant.

**Author Response:** We appreciate your suggestions.

**Reviewer Q10: **In the results there is mention of SALL4 nuclear staining. The magnifications shown are too low to see this in the figures presented. Perhaps include an offset at a higher magnification where you wish to demonstrate nuclear staining.

**Author Response:** Most of the pathological tissue samples stained for both nuclear and cytoplasm except for control tissue(dysgerminoma).

**Reviewer Q11: **For fascin to be acting as described in the discussion it would need to be staining more organized (with the actin cytoskeleton) rather than diffusely. Please relate your discussion items to your data more clearly.

**Author Response:** The present study was employed to identify the expression (Positive or negative) for fascin. Advanced techniques can be employed to identify its functional and precise location.

 (Reference: Klementieva, N. V., Snopova, L. B., Prodanets, N. N., Furman, O. E., Dudenkova, V. V., Zagaynova, E. V., Lukyanov, K. A., & Mishin, A. S. (2016). Fluorescence Imaging of Actin Fine Structure in Tumor Tissues Using SiR-Actin Staining. Anticancer research, 36(10), 5287–5294. https://doi.org/10.21873/anticanres.11100)

# Reviewer report: Reviewer response for version 4

**DOI:** 10.5256/f1000research.167388.r289189

**Published:** 2024-06-17

**Authors:** Sandhya Tamgadge

**Affiliations:** 1 D.Y. Patil University School of Dentistry, Navi Mumbai, Maharashtra, India

I have no further comments to make. The authors have diligently addressed the concerns which were  raised and incorporated the necessary revisions, resulting in a significantly improved and enriched manuscript. The revisions have enhanced the clarity and  depth, making it a valuable contribution to the field of oral pathology.

 While this study has provided valuable insights and advancements, there are still avenues for further exploration. Future research could focus on more  molecular markers as stated by authors to gain a more comprehensive understanding. The authors are encouraged to continue this  research  and build upon the foundation laid by this study.

Is the work clearly and accurately presented and does it cite the current literature?

Yes

If applicable, is the statistical analysis and its interpretation appropriate?

I cannot comment. A qualified statistician is required.

Are all the source data underlying the results available to ensure full reproducibility?

Yes

Is the study design appropriate and is the work technically sound?

Yes

Are the conclusions drawn adequately supported by the results?

Yes

Are sufficient details of methods and analysis provided to allow replication by others?

Yes

Reviewer Expertise:

Oral and maxillofacial pathology  (oral precancer cancer, odontogenic lesions, dental caries, oral infections, salivary gland lesions, oral developmental disorders), basic 3D medical animation.

I confirm that I have read this submission and believe that I have an appropriate level of expertise to confirm that it is of an acceptable scientific standard.

# Reviewer report: Reviewer response for version 4

**DOI:** 10.5256/f1000research.167388.r289192

**Published:** 2024-06-17

**Authors:** Pentti Nieminen

**Affiliations:** 1 Medical Informatics and Statistics Research Group, University of Oulu, Oulu, Finland

The statistical reporting has been improved. A few more points could be improved.

 1. I could not find any mention of the small number of cases in the discussion section. Please check whether the following sentences have been omitted from the manuscript: “However, in the present study the smaller sample size in odontogenic tumors and odontogenic cysts was one of the limitations. Therefore, for the better understanding of the dynamic and functional behavior of these molecules in the odontogenic lesions, studies on larger sample size and further experimental validation to elucidate their functional significance needs to be done.”

 2. A minor comment: The reporting of results could be improved by replacing p-value 1 with "p>0.999". P-value cannot be equal to 1.

Is the work clearly and accurately presented and does it cite the current literature?

No

If applicable, is the statistical analysis and its interpretation appropriate?

No

Are all the source data underlying the results available to ensure full reproducibility?

Partly

Is the study design appropriate and is the work technically sound?

No

Are the conclusions drawn adequately supported by the results?

Partly

Are sufficient details of methods and analysis provided to allow replication by others?

No

Reviewer Expertise:

Statistical reporting and data presentation

I confirm that I have read this submission and believe that I have an appropriate level of expertise to confirm that it is of an acceptable scientific standard, however I have significant reservations, as outlined above.

# Response

**Published:** 2024-06-22

**Authors:** Dr. Spoorti Kulkarni

**Affiliations:** Manipal College of Dental Sciences, Manipal Academy of Higher Education, Manipal, India

We are grateful to Pentti Nieminen for reviewing our article and offering valuable feedback.

 We have addressed the comments and have made revisions to the manuscript accordingly.

**Reviewer Q1.** I could not find any mention of the small number of cases in the discussion section. Please check whether the following sentences have been omitted from the manuscript: “However, in the present study the smaller sample size in odontogenic tumors and odontogenic cysts was one of the limitations. Therefore, for the better understanding of the dynamic and functional behavior of these molecules in the odontogenic lesions, studies on larger sample size and further experimental validation to elucidate their functional significance need to be done.”

**Author Response:** The following sentences have been added to the discussion.

 “In the current study, one of the limitations was the small sample size. Studies with a larger sample size are needed to understand the molecular (fascin and SALL4)  dynamics, functional behavior which is required for further validation and unravel the interaction of the pathways that activate these molecules. The interactivity of these molecules with other stem cell molecules in maintaining the stemness or pluripotency state of the cells needs to be explored.”

**Reviewer Q2.** A minor comment: The reporting of results could be improved by replacing p-value 1 with "p>0.999". P-value cannot be equal to 1.

**Author Response: **The p-value 1 has been replaced by “p>0.999” in Tables 1 and 2.

# Reviewer report: Reviewer response for version 3

**DOI:** 10.5256/f1000research.159983.r271942

**Published:** 2024-05-27

**Authors:** Sandhya Tamgadge

**Affiliations:** 1 D.Y. Patil University School of Dentistry, Navi Mumbai, Maharashtra, India

The authors have undertaken an impressive study, exhibiting concise yet captivating content, delving into the intricate realm of odontogenic lesions. Selection of markers exemplifies a thoughtful approach to this investigation. Nonetheless, the methodology section necessitates certain refinements as follows-

**Minor revisions-**

**Introduction **

1. Clarify if fascin and fascin-1 are synonymous terms which has been used.

**Methods**

1.  Resolve the discrepancy between the stated 46 odontogenic tumours samples and the 52 listed in the table. additionally providing descriptions of abbreviations S/NS.
2. Do mention all-control sample types used.

**RESULTS** –

1. verify the accuracy of the row entries for IRS, SALL4, and fascin in Tables 1 and 2.
2. Cite the specific IRS scoring method utilized, considering the multiple such methods are available in the literature .it will help future researchers.
3. Include few high magnification microphotographs to show cytoplasmic and nuclear staining.

**Discussion**-

1. The authors could enhance the study's value by sharing personal experiences and  overcoming difficulties faced, benefiting future researchers.

Overall, this well-executed study could be further strengthened by incorporating the above revisions to improve clarity and account for unique aspects of odontogenic lesions. The authors have produced an impressive body of work with room for refinement.

Is the work clearly and accurately presented and does it cite the current literature?

Yes

If applicable, is the statistical analysis and its interpretation appropriate?

I cannot comment. A qualified statistician is required.

Are all the source data underlying the results available to ensure full reproducibility?

Yes

Is the study design appropriate and is the work technically sound?

Yes

Are the conclusions drawn adequately supported by the results?

Yes

Are sufficient details of methods and analysis provided to allow replication by others?

Yes

Reviewer Expertise:

oral pathology ,oral precancer cancer,odontogenic lesions

I confirm that I have read this submission and believe that I have an appropriate level of expertise to confirm that it is of an acceptable scientific standard, however I have significant reservations, as outlined above.

# Response

**Published:** 2024-06-01

**Authors:** Dr. Spoorti Kulkarni

**Affiliations:** Manipal College of Dental Sciences, Manipal Academy of Higher Education, Manipal, India

We would like to thank   Dr. Sandhya Tamgadge for reviewing our article and providing us with valuable feedback. We have thoroughly reviewed the comments and made revisions to the manuscript accordingly. All the reviewer's comments have been addressed and corrected. We sincerely appreciate Dr. Sandhya for her time and consideration.

**Introduction**

 Q 1: Clarify if fascin and fascin-1 are synonymous terms which has been used.

  Response: The correction has been made as ‘fascin’ in the introduction.

**Methods**

 Q1: Resolve the discrepancy between the stated 46 odontogenic tumours samples and  the 52 listed in the table. additionally providing descriptions of abbreviations S/NS.

 Response: The discrepancy has been corrected in Table 1 and the abbreviations have been added for Table 1 and Table 2 in the revised version.

 Q2:Do mention all-control sample types used.

   Response: In the section of immunohistochemistry(IHC), we have mentioned all the positive control as well as internal controls for both the markers in the revised version.

**Results**

 Q1:verify the accuracy of the row entries for IRS, SALL4, and fascin in Tables 1 and 2.

 Response: Verification has been done and corrected in Table 1 and 2.

 Q1.Cite the specific IRS scoring method utilized, considering the multiple such methods are available in the literature .it will help future researchers.

  Response: Citation for IRS scoring method has been given as a modified version. (Ref: Klein M, Picard E, Vignaud JM, Marie B, Bresler L, Toussaint B, Weryha G, Duprez A, Leclere J. Vascular endothelial growth factor gene and protein: strong expression in thyroiditis and thyroid carcinoma. Journal of Endocrinology. 1999 Apr 1;161(1):41-50).

**Discussion-**

 Q1:The authors could enhance the study's value by sharing personal experiences and  overcoming difficulties faced, benefiting future researchers.

 Response: The main focus of the study was to understand one of the cellular processes in terms of motility and migration, which was elucidated by fascin positivity and SALL4 was employed for its stemness behavior. Advanced molecular techniques need to be employed to understand the dynamic behavior of these cells and the various crosstalks with other stem cell markers. Further, a larger sample is required for the validation of these markers.

 Q2:Include few high magnification microphotographs to show cytoplasmic and nuclear staining.

 Response: We have incorporated the same in the revised version.

# Reviewer report: Reviewer response for version 2

**DOI:** 10.5256/f1000research.154987.r203314

**Published:** 2023-09-07

**Authors:** Pentti Nieminen

**Affiliations:** 1 Medical Informatics and Statistics Research Group, University of Oulu, Oulu, Finland

1. The authors have been given many great suggestions during the previous review. They should have taken the time to process them and rewrite the manuscript. This revised manuscript is a bit disappointing.
2. Tables 1 and 2: You have compared the fascin and SALL 4 expressions using chi-square test. You have not stated this research question in the introduction or methods sections. Why to compare these distributions?
3. Statistical analysis sub-section: Please clearly state that you have presented the frequency distributions of the fascin and SALL 4 expressions by the sub-types of odontogenic tumors and cysts. Identify the main response variable (total IRS score ) here.
4. Tables 1 and 2: Please indicate in the SALL4 and fascin columns that you report frequencies. Please report also total sample size in the title. The use of statistical significance testing is not motivated in the introduction section and could be removed. The titles still need improvement.
5. The small number of cases in several subgroups of tumours and cysts is a limitation of your study. You have not addressed this in the discussion section.

Is the work clearly and accurately presented and does it cite the current literature?

No

If applicable, is the statistical analysis and its interpretation appropriate?

No

Are all the source data underlying the results available to ensure full reproducibility?

Partly

Is the study design appropriate and is the work technically sound?

No

Are the conclusions drawn adequately supported by the results?

Partly

Are sufficient details of methods and analysis provided to allow replication by others?

No

Reviewer Expertise:

Statistical reporting and data presentation

I confirm that I have read this submission and believe that I have an appropriate level of expertise to confirm that it is of an acceptable scientific standard, however I have significant reservations, as outlined above.

# Response

**Published:** 2023-12-09

**Authors:** Dr. Spoorti Kulkarni

**Affiliations:** Manipal College of Dental Sciences, Manipal Academy of Higher Education, Manipal, India

We thank Pentti Nieminen for reviewing our article and giving us valuable comments. We have carefully reviewed the comments and revised the manuscript accordingly. All comments pointed out by the reviewer have been corrected/revised, as below:

 Q1: Tables 1 and 2: You have compared the fascin and SALL4 expressions using the chi-square test. You have not stated this research question in the introduction or methods sections. Why to compare these distributions?

 Response: We have put forth the research question in the abstract, the comparison of fascin and SALL4 in the abstract (methodology section). However, we have revised the same in the introduction too.

 The literature search reveals a crosstalk between the pathway of these biomarkers (fascin and SALL4) which has been explained in the introduction. Hence an attempt was made to evaluate the expression as well as compare the expression of these markers in this study.

 Q2: Statistical analysis sub-section: Please clearly state that you have presented the frequency distributions of the fascin and SALL4 expressions by the sub-types of odontogenic tumors and cysts. Identify the main response variable (total IRS score) here.

 Response: Chi-square test was used to compare the frequency of distribution of categorised total IRS score with fascin and SALL4 in various odontogenic tumors (ameloblastoma and its histopathological variants, AOT) and odontogenic cysts (OKC, DC, COC, RC). The same has been incorporated in the results section.

 Q3: Tables 1 and 2: Please indicate in the SALL4 and fascin columns that you report frequencies. Please report also total sample size in the title. The use of statistical significance testing is not motivated in the introduction section and could be removed. The titles still need improvement.

 Response: There are various lesions and subtypes, therefore we have incorporated the same in the legends of Table 1 and Table 2.

 Q4: The small number of cases in several subgroups of tumours and cysts is a limitation of your study. You have not addressed this in the discussion section.

 Response: We have incorporated the limitations of the study in the discussion section.

 However, in the present study the smaller sample size in odontogenic tumors and odontogenic cysts was one of the limitation. Therefore, for the better understanding of the dynamic and functional behavior of these molecules in the odontogenic lesions, studies on larger sample size and further experimental validation to elucidate their functional significance needs to be done.

# Reviewer report: Reviewer response for version 1

**DOI:** 10.5256/f1000research.138468.r186991

**Published:** 2023-08-16

**Authors:** Qi-Wen Man

**Affiliations:** 1 Wuhan University, Wuhan, Hubei, China

I have carefully reviewed your research on the immunohistochemical expression of Fascin and SALL4 in ameloblastoma, adenomatoid tumor, and odontogenic cysts. The study aims to provide insights into these lesions by examining the expression of specific biomarkers. While the research direction is promising, there are several areas that require further attention to enhance the overall quality of the study:

1. The inclusion of multiple diseases as research subjects is commendable for its comprehensiveness. However, a critical concern lies in the lack of detailed characterization of each disease and their potential interrelationships. Providing a comprehensive description of their specific characteristics and how they relate to each other would enrich the study and offer a more robust foundation for future research.
2. The research would benefit from a more in-depth discussion of the developmental prospects of the selected biomarkers, Fascin, and SALL4. Additionally, it is essential to highlight the advantages of these biomarkers compared to other potential candidates.
3. While you have highlighted Fascin's involvement in the invasiveness of ameloblastoma, further elucidation is necessary regarding the reasons for its overexpression in Dentigerous cysts, Odontogenic Keratocyst, and Radicular cysts, or put forward corresponding conjectures. Similarly, the role of SALL4 in these dental pathologies requires more thorough exploration and explanation.
4. The absence of normal tissue as a control for IHC staining results is a notable concern. To strengthen the study's validity, it is crucial to include normal tissue controls or appropriate references for comparison.
5. The statistical tables used to present the IHC results require improvement. Employing more robust statistical methods, such as significance testing, to analyze the differences between lesions would enhance the analysis and provide more reliable conclusions.
6. Regarding the speculation on the functional roles of the two biological markers, it is suggested to conduct further experimental validation to elucidate their functional significance in the corresponding pathologies.
7. The background of the IHC staining was not the same which might influence the scores and analysis.

Is the work clearly and accurately presented and does it cite the current literature?

No

If applicable, is the statistical analysis and its interpretation appropriate?

Partly

Are all the source data underlying the results available to ensure full reproducibility?

Partly

Is the study design appropriate and is the work technically sound?

Yes

Are the conclusions drawn adequately supported by the results?

Partly

Are sufficient details of methods and analysis provided to allow replication by others?

Partly

Reviewer Expertise:

Immunohistochemistry, RNA sequencing, Odontogenic tumors

I confirm that I have read this submission and believe that I have an appropriate level of expertise to confirm that it is of an acceptable scientific standard, however I have significant reservations, as outlined above.

# Response

**Published:** 2023-09-05

**Authors:** Dr. Spoorti Kulkarni

**Affiliations:** Manipal College of Dental Sciences, Manipal Academy of Higher Education, Manipal, India

We thank Qi-Wen Man for reviewing our article and giving us valuable comments. I appreciate the time and efforts spent to make our work better. I have carefully reviewed the comments and revised the manuscript accordingly. All comments pointed out by the reviewer have been corrected.

 I have responded question wise.

 1: The inclusion of multiple diseases as research subjects is commendable for its comprehensiveness. However, a critical concern lies in the lack of detailed characterization of each disease and their potential interrelationships. Providing a comprehensive description of their specific characteristics and how they relate to each other would enrich the study and offer a more robust foundation for future research.

**Response: **In current study we have included Odontogenic tumors and cyst, which are pathology or disease arises from the odontogenic epithelium. Among the odontogenic tumors we have included, Ameloblastoma with its various histopathological variants (No:40),Adenomatiod odontogenic tumors (AOT) ( No:15),while odontogenic cyst includes 15 cases of each of Odontogenic cyst (OKC), Dentigerous cyst (DC), Radicular cyst (RC) and Calcifying odontogenic cyst( COC)(No:5).

 Literature review states among the odontogenic lesions Ameloblastoma and Odontogenic keratocyst are locally aggressive and recurrent lesions, also Ameloblastoma are the commonest and prevalent odontogenic tumor in Indian population is ameloblastoma which ranges from 14.02% to 71.4% when compared to other odontogenic tumors ^3,4^, while globally, pooled estimate of the incidence rate of ameloblastoma is 0.92 per million population per year ^5^. Among the odontogenic cyst we have highlighted only Odontogenic keratocyst, since these lesions are aggressive and recurrent compared to other cysts such as DC, a cyst associated with impacted tooth, RC, a cyst associated with caried or non-vital tooth, COC, a developmental cyst associated with calcification and ghost epithelial cells. We have incorporated the same in introduction.

 References:

 Ahire MS, Tupkari JV, Chettiankandy TJ, Thakur A, Agrawal RR. Odontogenic tumors: A 35-year retrospective study of 250 cases in an Indian (Maharashtra) teaching institute. Indian journal of cancer. 2018 Jul 1;55(3):265-72

 Pandiar D, Shameena PM, Sudha S, Varma S, Manjusha P, Banyal VS, Vijayan P. Odontogenic Tumors: A 13-year Retrospective Study of 395 Cases in a South Indian Teaching Institute of Kerala. Oral & Maxillofacial Pathology Journal. 2015 Jul 1;6(2)

 Hendra, F. N., Van Cann, E. M., Helder, M. N., Ruslin, M., de Visscher, J. G., Forouzanfar, T., & de Vet, H. C. W. Global incidence and profile of ameloblastoma: a systematic review and meta‐analysis. Oral Diseases. 2020 Jan;26(1):12-21

  Reichart P, Sciubba JJ, Philipsen HP. Splitters or lumpers: The 2017 WHO Classification of Head and Neck Tumors. J Am Dent Assoc. 2018;149:567-571.

 2: The research would benefit from a more in-depth discussion of the developmental prospects of the selected biomarkers, Fascin, and SALL4. Additionally, it is essential to highlight the advantages of these biomarkers compared to other potential candidates.

**Response: **Literature review confirms fascin contributes for cell motility and migration in many studies (Pubmed 376 articles), SALL4 contributes to stemness along with other stem cell markers ^8-13^ SOX2, OCT4, and NANOG. Studies have shown the expression of these stem cell markers in Ameloblastoma & OKC except SALL4. ^14 ^Most of the studies in SALL4 are related to malignant soft tissue tumors , ^15-16^ no reports are available of SALL4 expression in odontogenic lesions. Hence the present study aimed to evaluate the expression of fascin and SALL4 in odontogenic cysts & tumors.

 References:

  Matoba R, Niwa H, Masui S, Ohtsuka S, Carter MG, Sharov AA, Ko MS. Dissecting Oct3/4-regulated gene networks in embryonic stem cells by expression profiling. PloS one. 2006 Dec 20;1(1):e26.

  Nishimoto M, Fukushima A, Okuda A, Muramatsu M. The gene for the embryonic stem cell coactivator UTF1 carries a regulatory element which selectively interacts with a complex composed of Oct-3/4 and Sox-2. Molecular and cellular biology. 1999 Aug 1;19(8):5453-65.

  Wang J, Rao S, Chu J, Shen X, Levasseur DN, Theunissen TW, Orkin SH. A protein interaction network for pluripotency of embryonic stem cells. Nature. 2006 Nov 16;444(7117):364-8.

  Wu Q, Chen X, Zhang J, Loh YH, Low TY, Zhang W, Zhang W, Sze SK, Lim B, Ng HH. Sall4 interacts with Nanog and co-occupies Nanog genomic sites in embryonic stem cells. Journal of Biological Chemistry. 2006 Aug 25;281(34):24090-4.

  Zhang J, Tam WL, Tong GQ, Wu Q, Chan HY, Soh BS, Lou Y, Yang J, Ma Y, Chai L, Ng HH. Sall4 modulates embryonic stem cell pluripotency and early embryonic development by the transcriptional regulation of Pou5f1. Nature cell biology. 2006 Oct 1;8(10):1114-23.

  Zhou Q, Chipperfield H, Melton DA, Wong WH. A gene regulatory network in mouse embryonic stem cells. Proceedings of the National Academy of Sciences. 2007 Oct 16;104(42):16438-43.

 Phattarataratip E, Panitkul T, Khodkaew W, Anupuntanun P, Jaroonvechatam J, Pitarangsikul S. Expression of SOX2 and OCT4 in odontogenic cysts and tumors. Head & Face Medicine. 2021 Dec;17:1-7.

 Zhang X, Zhong N, Li X, Chen MB. TRIB3 promotes lung cancer progression by activating β-catenin signaling. European Journal of Pharmacology. 2019 Nov 15;863:172697

 Jiang G, Liu CT. Knockdown of SALL4 overcomes cisplatin-resistance through AKT/mTOR signaling in lung cancer cells. International Journal of Clinical and Experimental Pathology. 2018;11(2):634.

 3. While you have highlighted Fascin's involvement in the invasiveness of ameloblastoma, further elucidation is necessary regarding the reasons for its overexpression in Dentigerous cysts, Odontogenic Keratocyst, and Radicular cysts, or put forward corresponding conjectures. Similarly, the role of SALL4 in these dental pathologies requires more thorough exploration and explanation.

**Response: **We speculate the expression Fascin in cysts[(Dentigerous cysts(DC),Odontogenic keratocyst(OKC) and radicular cyst(RC)] could influence cell dynamics and focal adhesions. ^35^ We observed that the odontogenic epithelial cells were positive for SALL4 in the cytoplasm, stained diffusely, which we speculate could be in an inactive/dormant or mutant form which requires further investigation. Majority of OKC were devoid of SALL4 except in the basal cells. RC and DC, having marked infiltration of inflammatory cells had strong immune positivity for SALL4 in the cytoplasm, an interesting finding of this study. Hence the role of cytokines in stimulating SALL4 needs to be ruled out.

 Reference:

 Villari G, Jayo A, Zanet J, et al. A direct interaction between fascin and microtubules contributes to adhesion dynamics and cell migration. J Cell Sci. 2015;128(24):4601-4614.

 4:The absence of normal tissue as a control for IHC staining results is a notable concern. To

 strengthen the study's validity, it is crucial to include normal tissue controls or appropriate.

 references for comparison

**Response: **We have included the following under: Immunohistochemistry (IHC)

 Buccal mucosa tissue was used as positive control, the basal cells of the epithelium were stained positive and  endothelial cells with in the lesional tissue were internal controls for fascin antibody (Figure 1), while dysgerminoma was taken as a positive control for SALL4, bud and bell stage of tooth development was also included for the study(Figure 2).

 5.The statistical tables used to present the IHC results require improvement. Employing more robust statistical methods, such as significance testing, to analyze the differences between lesions would enhance the analysis and provide more reliable conclusions.

**Response: **Statistician was consulted for statistical analysis. The tables have been modified and have been uploaded in the OSF repository (S1, S2,S3,S4). In the main manuscript the tables are Table1, Table2.Regarding the evaluation of the statistical significance test, in all the odontogenic tumors, the staining intensity of fascin is similar compared to SALL4. Regarding the stained cell count, higher counts are observed with fascin as compared to SALL4.

  In relation to odontogenic cysts, viz odontogenic keratocyst and dentigerous cyst,the intensity of fascin staining was more than SALL4.Also the higher cell counts were observed in fascin as compared to SALL4 in odontogenic keratocyst. Fischer’s exact test was used to compare the distribution of staining intensity and cell count across SALL4 and fascin

 6. Regarding the speculation on the functional roles of the two biological markers, it is suggested to conduct further experimental validation to elucidate their functional significance in the corresponding pathologies.

**Response: **Thank you for your valuable advice. We will continue the experiments as an when we receive grants for further study.

 7.The background of the IHC staining was not the same which might influence the scores and analysis

**Response: **To eliminate the bias, two observers independently evaluated the expression of these biomarkers, selecting the most representative site separately under a light microscope at 200X and 400X magnification.

# Reviewer report: Reviewer response for version 1

**DOI:** 10.5256/f1000research.138468.r179066

**Published:** 2023-07-13

**Authors:** Konstantinos I Tosios

**Affiliations:** 1 Department of Oral Medicine and Pathology, School of Dentistry, National and Kapodistrian University of Athens, Athens, Greece

This is an immunohistochemical study on the expression of Fascin and SALL4 in ameloblastoma, adenomatoid tumor and odontogenic cysts. The manuscript could be considered for indexing, as it presents some new information, following full reconsideration of every sentence, as it is not properly written. The authors should carefully document the aim through the Introduction and state the aim clearly and concisely. In its present form the manuscript gives the impression that the authors applied two non-related antibodies in a convenient sample of odontogenic cysts and tumors, without having a working hypothesis to test. They should avoid the M&M critical mistakes, i.e., differences in positive controls among legends and texts - that alone could lead to a rejection. The Results and Discussion are not well structured, prohibiting their comprehension. All the above, coupled with the many grammatical errors, do not allow the (possible) significance of the findings to surface and make this manuscript, in its present form, not suitable for indexing.

****

**Major concerns**

- The Title refers to odontogenic cysts and tumors, but as it turns out the study includes two tumors and many different cysts. Why did the authors choose to examine those lesions?
- In the Introduction there is some information on three of the lesions investigated (what about DC, RC or COC?) and this is inconsistent, i.e., they present recurrence rate for OKC, but not for ameloblastoma or AOT. The authors should consistently present the same data for each lesion.
- Fascin and SALL4 seem to be two unrelated. Is there any association between them? Why did they choose to examine them? The authors use some references about the role in malignant neoplasms, however the lesions under consideration are not malignant neoplasms, while most of the references do not include studies on their expression in malignant neoplasms. Please carefully check your references.
- The aim of the study should be concise and clear. It should be reconsidered.
- M&M: “Before the start of the study, statistician was consulted and based on the literature review, availability of the material in the archives of the department and the availability of the budget the sample was decided”. Are 6 AOTs or 5 COC’s an adequate sample size?
- The Patients and Tissues samples section needs restructuring. Origin of material, inclusion/exclusion criteria, IRB etc.
- What was the positive control for Fascin, the basal cells of the oral epithelium (Figure) or the endothelial cells of the buccal mucosa (text)? Any supporting reference of any of them?
- In general, please present the findings in a more orderly manner, i.e., what did you see in the positive controls (membranous and cytoplasmic expression in the basal cells of the epithelium, etc.).
- Follow the same order of the antibodies, i.e., if you choose Fascin/SALL4 then present the results first for fascin and then for SALL4.
- “40 cases of ameloblastoma variants from the year 2012 to 2017”. This information is not proper here.
- Results should be rewritten in a more orderly manner.
- Discussion: “Researchers have worked on…”. There are no references to support this.
- Discussion: “the odontogenic epithelial cells were positive for SALL4 in the cytoplasm, stained diffusely, which we speculate could be in an inactive/dormant or mutant form which requires further investigation”. Is there any literature supporting that cytoplasmic expression in not a cross reaction with another protein?
- The last paragraph of the Discussion is interesting but lacks connections with the rest of the Discussion.

**Minor considerations:**

- “Are said to”: this is not an “mouth to ear” information, it is well established in the literature.
- “for its local but aggressive biological behavior”: What do they mean by “local” behavior?
- “are benign with less recurrence”. What is “less” recurrence?
- Please check the classification of ameloblastomas, it is not correct. Furthermore, in the M&M, ameloblastomas are subclassified based on their histopathological features, but there in no mention to them in the Intro.
- “fascin might contribute for the local migratory behavior of these odontogenic cells”. Which cells?
- “The document of the protocol has been uploaded in the repository (Open Science Framework protocol.io) ^26^ and hydrated”. Was the protocol hydrated? Please carefully check the text throughout the manuscript.
- “incubated with primary antibodies (rabbit monoclonal IgG for SALL4 and mouse monoclonal IgG1 for fascin) ^26^ against fascin (clone SC-21743, Santa Cruz Biotechnology USA, Inc) diluted 1:200”. Please correct.
- “To eliminate bias, two observers independently evaluated the expression of these, selecting the most representative site separately under a light microscope...” This information should not be presented here.
- “Odontogenic tumors, AOT and developmental odontogenic cysts, COC”. Isn’t OKC a developmental cyst?

Is the work clearly and accurately presented and does it cite the current literature?

Partly

If applicable, is the statistical analysis and its interpretation appropriate?

I cannot comment. A qualified statistician is required.

Are all the source data underlying the results available to ensure full reproducibility?

Yes

Is the study design appropriate and is the work technically sound?

Partly

Are the conclusions drawn adequately supported by the results?

No

Are sufficient details of methods and analysis provided to allow replication by others?

Yes

Reviewer Expertise:

Oral pathology

I confirm that I have read this submission and believe that I have an appropriate level of expertise to state that I do not consider it to be of an acceptable scientific standard, for reasons outlined above.

# Response

**Published:** 2023-09-05

**Authors:** Dr. Spoorti Kulkarni

**Affiliations:** Manipal College of Dental Sciences, Manipal Academy of Higher Education, Manipal, India

We thank Konstantinos I. Tosios for reviewing our article and giving us your valuable comments. To the best of my ability I have tried to answer the queries and revised the manuscript accordingly.

 I have responded question wise.

 Q: The Title refers to odontogenic cysts and tumors, but as it turns out the study includes two tumors and many different cysts. Why did the authors choose to examine those lesion?

**Response: **Literature review states among the odontogenic lesions Ameloblastoma and Odontogenic keratocyst are locally aggressive and recurrent lesions, also the commonest and prevalent odontogenic tumor in Indian population is ameloblastoma which ranges from 14.02% to 71.4% when compared to other odontogenic tumors ^3,4^,while globally, pooled estimate of the incidence rate of ameloblastoma is 0.92 per million population per year. ^5^The archives of the department did have other odontogenic lesions which were taken up for study

 References:

 Ahire MS, Tupkari JV, Chettiankandy TJ, Thakur A, Agrawal RR. Odontogenic tumors: A 35-year retrospective study of 250 cases in an Indian (Maharashtra) teaching institute. Indian journal of cancer. 2018 Jul 1;55(3):265-72

 Pandiar D, Shameena PM, Sudha S, Varma S, Manjusha P, Banyal VS, Vijayan P. Odontogenic Tumors: A 13-year Retrospective Study of 395 Cases in a South Indian Teaching Institute of Kerala. Oral & Maxillofacial Pathology Journal. 2015 Jul 1;6(2)

 Hendra, F. N., Van Cann, E. M., Helder, M. N., Ruslin, M., de Visscher, J. G., Forouzanfar, T., & de Vet, H. C. W. Global incidence and profile of ameloblastoma: a systematic review and meta‐analysis. Oral Diseases. 2020 Jan;26(1):12-21

 Q: In the Introduction there is some information on three of the lesions investigated (what about DC, RC or COC?) and this is inconsistent, i.e., they present recurrence rate for OKC, but not for ameloblastoma or AOT. The authors should consistently present the same data for each lesion.

**Response: **Among the odontogenic cyst we have highlighted only Odontogenic keratocyst, since these lesions are aggressive and recurrent compared to other cysts such as DC, RC or COC, we have incorporated the same in introduction. We have also modified the same for Ameloblastoma as well, in ‘Introduction’: ‘The recurrence varies among various populations, 9.8% according to a Chinese study, ^6^while in European multicenter study it is reported to be 19.3%, ^7^ also tumors larger than 6 cm and involving the soft tissues or adjacent anatomical structures are associated with early recurrence irrespective of method of surgery. Also, conservatively (marsupialization, enucleation, curettage) treated cases have a high recurrence rate compared to radical treatment. ^1^ however there is no concrete data pertaining recurrence on AOT

 References:

 Yang R, Liu Z, Gokavarapu S, Peng C, Ji T, Cao W. Recurrence and cancerization of ameloblastoma: multivariate analysis of 87 recurrent craniofacial ameloblastoma to assess risk factors associated with early recurrence and secondary ameloblastic carcinoma. Chinese Journal of Cancer Research. 2017 Jun;29(3):189.

  Boffano P, Cavarra F, Tricarico G, Masu L, Brucoli M, Ruslin M, Forouzanfar T, Ridwan-Pramana A, Rodríguez-Santamarta T, Ranz MR, de Vicente JC. The epidemiology and management of ameloblastomas: A European multicenter study. Journal of Cranio-Maxillofacial Surgery. 2021 Dec 1;49(12):1107-12.].

 Q: Fascin and SALL4 seem to be two unrelated. Is there any association between them? Why did they choose to examine them? The authors use some references about the role in malignant neoplasms, however the lesions under consideration are not malignant neoplasms, while most of the references do not include studies on their expression in malignant neoplasms. Please carefully check your references

**Response: **Literature review confirms fascin contributes for cell motility and migration in many studies (Pubmed 376 articles), SALL4 contributes to stemness along with other stem cell markers (SOX2, OCT4, and NANOG). ^8-13^ Studies have shown the expression of these stem cell markers in Ameloblastoma & OKC except SALL4. ^14^ Most of the studies in SALL4 are related to malignant soft tissue tumors, ^15,16^ no reports are available of SALL4 expression in odontogenic lesions. Hence the present study aimed to evaluate the expression of fascin and SALL4 in odontogenic cysts & tumors.

 References:

 Matoba R, Niwa H, Masui S, Ohtsuka S, Carter MG, Sharov AA, Ko MS. Dissecting Oct3/4-regulated gene networks in embryonic stem cells by expression profiling. PloS one. 2006 Dec 20;1(1):e26.

 Nishimoto M, Fukushima A, Okuda A, Muramatsu M. The gene for the embryonic stem cell coactivator UTF1 carries a regulatory element which selectively interacts with a complex composed of Oct-3/4 and Sox-2. Molecular and cellular biology. 1999 Aug 1;19(8):5453-65.

 Wang J, Rao S, Chu J, Shen X, Levasseur DN, Theunissen TW, Orkin SH. A protein interaction network for pluripotency of embryonic stem cells. Nature. 2006 Nov 16;444(7117):364-8.

 Wu Q, Chen X, Zhang J, Loh YH, Low TY, Zhang W, Zhang W, Sze SK, Lim B, Ng HH. Sall4 interacts with Nanog and co-occupies Nanog genomic sites in embryonic stem cells. Journal of Biological Chemistry. 2006 Aug 25;281(34):24090-4.

 Zhang J, Tam WL, Tong GQ, Wu Q, Chan HY, Soh BS, Lou Y, Yang J, Ma Y, Chai L, Ng HH. Sall4 modulates embryonic stem cell pluripotency and early embryonic development by the transcriptional regulation of Pou5f1. Nature cell biology. 2006 Oct 1;8(10):1114-23.

  Zhou Q, Chipperfield H, Melton DA, Wong WH. A gene regulatory network in mouse embryonic stem cells. Proceedings of the National Academy of Sciences. 2007 Oct 16;104(42):16438-43.

 Phattarataratip E, Panitkul T, Khodkaew W, Anupuntanun P, Jaroonvechatam J, Pitarangsikul S. Expression of SOX2 and OCT4 in odontogenic cysts and tumors. Head & Face Medicine. 2021 Dec;17:1-7.

 Zhang X, Zhong N, Li X, Chen MB. TRIB3 promotes lung cancer progression by activating β-catenin signaling. European Journal of Pharmacology. 2019 Nov 15;863:172697

 Jiang G, Liu CT. Knockdown of SALL4 overcomes cisplatin-resistance through AKT/mTOR signaling in lung cancer cells. International Journal of Clinical and Experimental Pathology. 2018;11(2):634.

 We agree on the fact that Fascin and SALL4 seems to be two unrelated molecules. SALL4 is activated by various pathways such as Wnt/β-catenin, ^15 ^PI3K/AKT, signalling pathway through targeting PTEN ^16^or Notch signalling pathway ^17^, thus facilitating migration, invasion and proliferation, while Fascin is activated via PI3K/ Akt pathway. ^18^Also, literature reports cross talk between Wnt/β-catenin and PI3K/ Akt pathways or simultaneous activation of these pathways contributing for proliferation and cell migration. ^19-21^ Hence we have made an attempt to study the expression of these two markers.

 References:

 Zhang X, Zhong N, Li X, Chen MB. TRIB3 promotes lung cancer progression by activating β-catenin signaling. European Journal of Pharmacology. 2019 Nov 15;863:172697

 Jiang G, Liu CT. Knockdown of SALL4 overcomes cisplatin-resistance through AKT/mTOR signaling in lung cancer cells. International Journal of Clinical and Experimental Pathology. 2018;11(2):634.

 Park JT, Chen X, Tropè CG, Davidson B, Shih IM, Wang TL. Notch3 overexpression is related to the recurrence of ovarian cancer and confers resistance to carboplatin. The American journal of pathology. 2010 Sep 1;177(3):1087-94.

 Fleming-de-Moraes CD, Rocha MR, Tessmann JW, de Araujo WM, Morgado-Diaz JA. Crosstalk between PI3K/Akt and Wnt/β-catenin pathways promote colorectal cancer progression regardless of mutational status. Cancer biology & therapy. 2022 Dec 31;23(1):1-3.

 Laguë MN, Paquet M, Fan HY, Kaartinen MJ, Chu S, Jamin SP, Behringer RR, Fuller PJ, Mitchell A, Doré M, Huneault LM. Synergistic effects of Pten loss and WNT/CTNNB1 signaling pathway activation in ovarian granulosa cell tumor development and progression. Carcinogenesis. 2008 Nov 1;29(11):2062-72.

 Deming DA, Leystra AA, Nettekoven L, Sievers C, Miller D, Middlebrooks M, Clipson L, Albrecht D, Bacher J, Washington MK, Weichert J. PIK3CA and APC mutations are synergistic in the development of intestinal cancers. Oncogene. 2014 Apr;33(17):2245-54.

 Pearson HB, Phesse TJ, Clarke AR. K-ras and Wnt signaling synergize to accelerate prostate tumorigenesis in the mouse. Cancer research. 2009 Jan 1;69(1):94-101.

 Q: The aim of the study should be concise and clear. It should be reconsidered.

**Response: **The aim of the present study was to evaluate the expression of fascin and SALL4 in histopathological variants of ameloblastoma, AOT and various odontogenic cysts namely OKC, Dentigerous cyst and radicular cyst. Following a thorough literature search we hypothesise that fascin might contribute for the local migratory behaviour of the odontogenic tumors and cysts while SALL4 may contribute to stemness property.

 M&M: “Before the start of the study, statistician was consulted and based on the literature review, availability of the material in the archives of the department and the availability of the budget the sample was decided”. Are 6 AOTs or 5 COC’s an adequate sample size?

**Response:** The small number of cases in the present study regarding odontogenic tumors and its subgroup as well as in odontogenic cysts were limitation of this study. Further studies with large sample size is required for the better understanding of these biomarkers in these lesions.

 Q: The Patients and Tissues samples section needs restructuring. Origin of material, inclusion/exclusion criteria, IRB etc.

**Response:** Samples were retrieved from the Department of Oral and Maxillofacial Pathology, Manipal College of Dental Sciences, Manipal, India after the approval from the Institutional Ethical Committee. This study was approved (IEC approval number 360/2019, 14-05-2019; IEC 156/2014, 12-03-2014) by the Institutional Ethical Committee, Kasturba Medical College, Manipal Academy of Higher Education, Manipal, India. Participant consent was waived by the committee.

 The samples taken up for the study did comply to inclusion and exclusion criteria which included histologically diagnosed cases of odontogenic cysts and tumors from 2012-2017. All the samples taken for the current study were prior to the patient receiving any treatment, cases with recurrence were excluded. The diagnosis of the above said odontogenic cysts and tumors were done based on clinical and histological features (using H&E staining) according to WHO guidelines.

 Q:What was the positive control for Fascin, the basal cells of the oral epithelium (Figure) or the endothelial cells of the buccal mucosa (text)? Any supporting reference of any of them

**Response: **Buccal mucosa tissue ^22^ ( Figure 1), was considered as external positive control while endothelial cells with in the samples to be tested was considered for internal positive control for Fascin expression. The reference article is as below.

 Rodrigues PC, Sawazaki-Calone I, Ervolino de Oliveira C, et al. Fascin promotes migration and invasion and is a prognostic marker for oral squamous cell carcinoma. Oncotarget. 2017;8(43):74736-74754.

 Q:In general, please present the findings in a more orderly manner, i.e., what did you see in the positive controls (membranous and cytoplasmic expression in the basal cells of the epithelium, etc.)

**Response: **For fascin, the positive control used was buccal mucosa, the cytoplasm of the basal cells of the epithelium were stained positive. In case of SALL4 dysgerminoma was taken as positive control where in nuclear expression in the cells is seen.

 Q:Follow the same order of the antibodies, i.e., if you choose Fascin/SALL4 then present the results first for fascin and then for SALL4.

**Response:** Correction is done as follows:

 Immunohistochemically stained sections of various odontogenic cysts and tumor tissue were evaluated for expression of fascin in the cell membrane, between cell boundaries and cytoplasm of peripheral ameloblastic cells, stellate reticulum like cells and stromal cells of 40 cases of ameloblastoma variants while expression of SALL4 was observed in the cytoplasm as well as nuclei of peripheral ameloblastic cells and stellate reticulum like cells. The total IRS score was the main outcome (Table 1, Table 2). The expression of fascin and SALL4 varied from case to case as well as in the same tissue section. Most of the variants of ameloblastoma were strongly positive for fascin but cases of desmoplastic ameloblastoma (5/5) were negative for fascin

 (Figure 1D). Fascin expression was found to be weak or absent in stellate reticulum like cells (Figure 1). In cases of unicystic ameloblastoma, positivity for fascin was observed in the basal as well as in the suprabasal layers (Figure 1C). However intra-group comparison did not show any significant difference. AOT was immune-positive to fascin in few areas (< 25%) with mild to moderate intensity (Figure 1E). Fascin expression in odontogenic cysts (OKC, RC, DC) (Figure 1G–I) was strongly positive with greater than 75% cells, while intensity ranged from moderate to strong along the cystic lining. COC revealed immune positivity ranging from 25-50% (Figure 1F). The SALL4 positivity was heterogeneous with varied intensity and staining pattern. In most of the histopathological variant of ameloblastoma, the immunopositivity observed, was diffuse in the cytoplasm and less localised to the nucleus (Figure 2A–C). The stromal cells were devoid of its expression except in the endothelial cells. SALL4 expression in odontogenic cysts was strongly positive with greater than 75% cells exhibiting diffuse cytoplasmic staining. Nuclear staining was evident in few cells (Figure 2E–G). COC was immune-negative (Figure 2H).

 Regarding the evaluation of the statistical significance test, in all the odontogenic tumors, the staining intensity of fascin is similar compared to SALL4. With regard to the stained cell count, higher counts are observed with fascin as compared to SALL4. In relation to odontogenic cysts, odontogenic keratocyst and dentigerous cyst,the intensity of fascin is more than SALL4.Also the higher cell counts were observed in fascin as compared to SALL4 in odontogenic keratocyst. (S1,S2,S3,S4,Table1,Table2)

 Q:Results should be rewritten in a more orderly manner.

**Response:** Correction has been made

 Q:40 cases of ameloblastoma variants from the year 2012 to 2017”. This information is not proper here.

**Response:** The above sentence has been removed as instructed.

 Q:Discussion: “Researchers have worked on…”. There are no references to support this

**Response: **The reference article for support, Ref no 23: Fuchigami T, Ono Y, Kishida S, Nakamura N.Jpn Dent Sci Rev. 2021 Nov;57:27-32. : Molecular biological findings of ameloblastoma.

 Q:Discussion: “the odontogenic epithelial cells were positive for SALL4 in the cytoplasm, stained diffusely, which we speculate could be in an inactive/dormant or mutant form which requires further investigation”. Is there any literature supporting that cytoplasmic expression in not a cross reaction with another protein?

**Response: **Studies pertaining to immunogenicity of SALL4 oncogenic protein-derived peptides, especially A18 K and R18 A peptides have been reported, however this needs to be validated in odontogenic tumors.

 Kroemer, M., Spehner, L., Mercier-Letondal, P., Boullerot, L., Kim, S., Jary, M., Galaine, J., Picard, E., Ferrand, C., Nguyen, T., Larosa, F., Adotévi, O., Godet, Y., & Borg, C.SALL4 oncogene is an immunogenic antigen presented in various HLA-DR contexts. *Oncoimmunology*.2018: *7*(4);e1412030.

 Q: The last paragraph of the Discussion is interesting but lacks connections with the rest of the Discussion.

**Response: **The origin of odontogenic cysts and tumors are from the odontogenic apparatus. Hence an attempt was made to study the expression of these biomarkers in pre and post stages of tooth germ.

**Minor considerations:**

 Q:Are said to”: this is not an “mouth to ear” information, it is well established in the literature

**Response: **The sentence has been corrected as, ‘ Odontogenic cysts and tumors originates from odontogenic apparatus or oral epithelium’

 Q: “for its local but aggressive biological behavior”: What do they mean by “local” behavior?

**Response: **The term ‘local’ refers to localized region of the Jaws

 Q:are benign with less recurrence”. What is “less” recurrence

**Response: **The sentence has been modified to ‘ Adenomatoid odontogenic tumors (AOT) are benign with rare recurrence.

 Q:Please check the classification of ameloblastomas, it is not correct. Furthermore, in the M&M, ameloblastomas are subclassified based on their histopathological features, but there in no mention to them in the Intro

**Response: **Due to word limitations in introduction we have incorporated the histopathological variants in M&M.

 Q:fascin might contribute for the local migratory behavior of these odontogenic cells”. Which cells?

**Response: **Fascin might contribute for the local migratory behavior of the odontogenic epithelial cells of Ameloblastoma, AOT, OKC,RC,DC,COC.

 Q:The document of the protocol has been uploaded in the repository (Open Science Framework protocol.io) ^26^ and hydrated”. Was the protocol hydrated? Please carefully check the text throughout the manuscript.

**Response: **This has been an oversight, we have corrected as follows

 Immunohistochemical staining of the tissue sections from each of the cases selected was done using the streptavidin-biotin method. In brief, 4 ��m sections were mounted on 3-aminopropyltriethoxysilane (APES) coated slides (Novolink Polymer Detection System, Novocastra). Sections were then deparaffinized in xylene, which is done in three grades for 10 minutes and hydrated in different grades of alcohol (ranging from absolute alcohol (10 minutes), 95 % alcohol (10 minutes), 70% (10 minutes), 50% (10 minutes) each). Sections were then incubated with primary antibodies,rabbit antihuman SALL4 monoclonal antibody at a dilution of 1:100(IgG, clone EP-299, PathnSitu, Livermore, USA ),mouse antihuman fascin monoclonal antibody ( IgG1,clone55K-2, SC-21743, Santa Cruz Biotechnology USA, Inc) diluted at 1:200. The sections were subsequently washed in tris-buffered saline and incubated with secondary biotinylated antibody and streptavidin-biotin peroxidase complex (Novolink Polymer Detection System, Novocastra) for 30 minutes each. Diaminobenzidine (DAB) was used as the chromogen and the sections were counterstained with Mayer’s hematoxylin. Buccal mucosa tissue was used as positive control and endothelial cells were internal controls for fascin antibody ( Figure 1), while dysgerminoma was taken as a positive control, bud and bell stage of tooth development were also included for the expression of SALL4 ( Figure 2). The primary antibody was replaced during IHC staining for the negative control as per standard immunohistochemical protocol. The document of the protocol has been uploaded in the repository (Open Science Framework protocol.io)48

 Q:“incubated with primary antibodies (rabbit monoclonal IgG for SALL4 and mouse monoclonal IgG1 for fascin) ^26^ against fascin (clone SC-21743, Santa Cruz Biotechnology USA, Inc) diluted 1:200”. Please correct.

**Response: **We corrected the same as follows: 

 Sections were then incubated with primary antibodies,rabbit antihuman SALL4 monoclonal antibody at a dilution of 1:100(IgG, clone EP-299, PathnSitu, Livermore, USA ),mouse antihuman fascin monoclonal antibody ( IgG1,clone55K-2, SC-21743, Santa Cruz Biotechnology USA, Inc) diluted to 1:200

 Q:“To eliminate bias, two observers independently evaluated the expression of these, selecting the most representative site separately under a light microscope...” This information should not be presented here

**Response: **We have removed the sentence from results and incorporated in ‘Immuno staining evaluation’.

 Immunostaining evaluation

 Presence of brown color at the end of staining was considered as positive reactivity. The slides were evaluated with a light microscope (Olympus BX41) attached with Olympus DP20 microscope camera (Olympus Singapore Pvt Ltd, Singapore) at 20× & 40× magnification. The distribution of antibodies was assessed in the cytoplasm and cell membrane of ameloblastic lining of the lesions for fascin while SALL4 staining was evaluated in nuclear and cytoplasmic areas. In each case, three fields were randomly selected, and two observers independently evaluated the expression of these, selecting the most representative site separately under a light microscope at 200× and 400× magnification.

 Q:Odontogenic tumors, AOT and developmental odontogenic cysts, COC”. Isn’t OKC a developmental cyst?

**Response: **Yes, OKC, dentigerous are developmental while radicular cyst is inflammatory in origin

# Reviewer report: Reviewer response for version 1

**DOI:** 10.5256/f1000research.138468.r171400

**Published:** 2023-05-16

**Authors:** Pentti Nieminen

**Affiliations:** 1 Medical Informatics and Statistics Research Group, University of Oulu, Oulu, Finland

In this study, the expression of SALL4 and fascin were evaluated in odontogenic tumours and cysts. The findings of the study on fascin expression clarify their role in motility and localized invasion or maintenance of cellular homeostasis. However, the expression of SALL4 remains unclear.

 The focus of this review is mainly on data reporting. My comments to the authors are as follows:

1. One positive aspect of your manuscript was that it was a short report, which kept the focus on the main objective.
2. I enjoyed reading the introduction section of this manuscript. The research question related to was well described.
3. Introduction, last sentence: Please consider specifying the aims of your study. It would help your readers if you stated that the aim was to estimate the prevalence of two biomarkers, SALL4 and fascin, in histopathological variants of ameloblastoma, AOT and various odontogenic cysts. In addition, your aim was to compare the prevalence of biomarkers between different tumours and cysts.
4. As a short report, the statistical intensity of the manuscript was lower than the average of articles published in visible general medical journals. However, the quality of statistical reporting and data presentation was weak: I gave it a score of 3 on a scale of 0 (poor) to 10 (very high). In particular, the quality of data presentation in tables should be improved.
5. Consider including a table describing the distributions of the basic characteristics of the cases, their clinical history, and the main outcome variables. If many characteristics of human cases are described, the details of the cases and the number of cases contributing to the analysis are best included in a tabular presentation.
6. Tables 1A and 1B are not well prepared. Tables with proper titles, clear labelling and optimally presented data will enable readers to scrutinise the data. The tables did not have clear titles. The current formatting of these tables resembled a spreadsheet, and the lines of the same size between each row and column did not help to clarify the different data presented in the tables. Not all abbreviations were defined. What differences do the statistical significance tests evaluate?
7. Statistical analysis section should help a knowledgeable reader to judge the appropriateness of the methods for the study and to verify the methods reported. You should improve the description of statistical methods. State clearly that total staining scores of SALL4 and fascin are your main outcome measures. Then describe that the prevalence of these response variables was estimated in different tumour and cyst groups. Clarify the variables and methods for each significance test performed in the study.
8. The small number of cases in several subgroups of tumours and cysts is a limitation of your study. Please address this in the discussion section.

Is the work clearly and accurately presented and does it cite the current literature?

No

If applicable, is the statistical analysis and its interpretation appropriate?

No

Are all the source data underlying the results available to ensure full reproducibility?

Partly

Is the study design appropriate and is the work technically sound?

No

Are the conclusions drawn adequately supported by the results?

Partly

Are sufficient details of methods and analysis provided to allow replication by others?

No

Reviewer Expertise:

Statistical reporting and data presentation

I confirm that I have read this submission and believe that I have an appropriate level of expertise to confirm that it is of an acceptable scientific standard, however I have significant reservations, as outlined above.

# Response

**Published:** 2023-09-05

**Authors:** Dr. Spoorti Kulkarni

**Affiliations:** Manipal College of Dental Sciences, Manipal Academy of Higher Education, Manipal, India

We thank Pentti Nieminen for reviewing our article and giving us valuable comments. I appreciate the time and efforts spent to make our work better. I have carefully reviewed the comments and revised the manuscript accordingly. All comments pointed out by the reviewer have been corrected.

 I have responded question wise.

 3. Introduction, last sentence: Please consider specifying the aims of your study. It would help your readers if you stated that the aim was to estimate the prevalence of two biomarkers, SALL4 and fascin, in histopathological variants of ameloblastoma, AOT and various odontogenic cysts. In addition, your aim was to compare the prevalence of biomarkers between different tumours and cysts.

**Response: **We have explained the same in the abstract and didn’t want to repeat the same in the introduction. However as advised we have modified the same in the introduction. Also from the oral pathologist view prevalence is synonym to expression and estimate is synonym to evaluate, we have incorporated the same in introduction. Thus, the aim of the present study was to evaluate the expression of these two biomarkers fascin and SALL4 in histopathological variants of ameloblastoma, AOT and various odontogenic cysts namely Odontogenic keratocyst, Dentigerous cyst and Radicular cyst. Since the biological behaviour of cyst and tumors differs, we didn’t do the comparison between them.

 4. As a short report, the statistical intensity of the manuscript was lower than the average of articles published in visible general medical journals. However, the quality of statistical reporting and data presentation was weak: I gave it a score of 3 on a scale of 0 (poor) to 10 (very high). In particular, the quality of data presentation in tables should be improved.

**Response:** We have revised the data presentation in the Tables 1 and Table 2 (other tables have been added as supplementary files S1, S2,S3,S4).

 5. Consider including a table describing the distributions of the basic characteristics of the cases, their clinical history, and the main outcome variables. If many characteristics of human cases are described, the details of the cases and the number of cases contributing to the analysis are best included in a tabular presentation.

**Response:** The excel sheet with the clinical details of the patients has been attached as a supplementary file in the OSF repository.

 6.Tables 1A and 1B are not well prepared. Tables with proper titles, clear labelling and optimally presented data will enable readers to scrutinise the data. The tables did not have clear titles. The current formatting of these tables resembled a spreadsheet, and the lines of the same size between each row and column did not help to clarify the different data presented in the tables. Not all abbreviations were defined. What differences do the statistical significance tests evaluate?

**Response:** The tables have been modified as per reviewers comments and have been uploaded in the OSF repository. Regarding the evaluation of the statistical significance test, in all the odontogenic tumors, the staining intensity of fascin is similar compared to SALL4. With regard to the stained cell count, higher counts are observed with fascin as compared to SALL4.

 In relation to odontogenic cysts viz odontogenic keratocyst and dentigerous cyst, the intensity of fascin staining is more than SALL4.Also the higher cell counts were observed in fascin as compared to SALL4 in odontogenic keratocyst.

 7. Statistical analysis section should help a knowledgeable reader to judge the appropriateness of the methods for the study and to verify the methods reported. You should improve the description of statistical methods. State clearly that total staining scores of SALL4 and fascin are your main outcome measures. Then describe that the prevalence of these response variables was estimated in different tumour and cyst groups. Clarify the variables and methods for each significance test performed in the study.

**Response:**  We have uploaded the modified tables and these tables are self-explanatory, as per the advice of the reviewer. Fischers exact test was used to compare the distribution of staining intensity and cell count across SALL4 and Fascin.

 8. The small number of cases in several subgroups of tumours and cysts is a limitation of your study. Please address this in the discussion section.

**Response:** The small number of cases in the present study with regard to odontogenic tumors and its subgroup as well as in odontogenic cysts were limitation of this study. Further studies with large sample size is required for the better understanding of these biomarkers in these lesions.
